# A quantum resistance memristor for an intrinsically traceable International System of Units standard

**DOI:** 10.1038/s41565-025-02037-5

**Published:** 2025-10-27

**Authors:** Gianluca Milano, Xin Zheng, Fabio Michieletti, Giuseppe Leonetti, Gabriel Caballero, Ilker Oztoprak, Luca Boarino, Özgür Bozat, Luca Callegaro, Natascia De Leo, Isabel Godinho, Daniel Granados, Itir Koymen, Mariela Menghini, Enrique Miranda, Luís Ribeiro, Carlo Ricciardi, Jordi Suñe, Vitor Cabral, Ilia Valov

**Affiliations:** 1 Advanced Materials Metrology and Life Sciences Division, INRiM (Istituto Nazionale di Ricerca Metrologica), Turin, Italy; 2 Forschungszentrum Jülich, Peter Grünberg Institute (PGI-7), Jülich, Germany; 3 Department of Applied Science and Technology, Politecnico di Torino, Turin, Italy; 4 IMDEA Nanociencia, Cantoblanco, Madrid, Spain; 5 Facultad de Ciencias Físicas, Universidad Complutense de Madrid, Madrid, Spain; 6 Department of Physics, Gebze Technical University, Gebze, Kocaeli Turkey; 7 TUBITAK National Metrology Institute (UME), Gebze, Turkey; 8 Quantum Metrology and Nanotechnologies Division, INRiM (Istituto Nazionale di Ricerca Metrologica), Turin, Italy; 9 Instituto Português da Qualidade, Caparica, Portugal; 10 Department of Electrical and Electronics Engineering, TOBB University of Economics and Technology, Ankara, Turkey; 11 Departament d’Enginyeria Electrònica, Universitat Autònoma de Barcelona, Cerdanyola del Valles, Spain; 12 Institute of Electrochemistry and Energy Systems, Bulgarian Academy of Sciences (BAS), Sofia, Bulgaria

**Keywords:** Electronic devices, Electronic and spintronic devices

## Abstract

The recent revision of the International System of Units (SI)—which fixed the numerical values of nature’s fundamental constants—has opened new perspectives for practical realizations of SI units. Here we demonstrate an intrinsic resistance standard based on memristive nanoionic cells that operate in air at room temperature and are directly accessible to end users. By driving these devices into the quantum conductance regime and using an electrochemical-polishing-based programming strategy, we achieved quantum conductance levels that can be exploited as intrinsic standard values. An interlaboratory comparison confirmed metrological consistency, with deviations of –3.8% and 0.6% from the agreed SI values for the fundamental quantum of conductance, *G*_0_, and 2*G*_0_, respectively. These results lay the groundwork for the implementation of national metrology institute services on chip and for the development of self-calibrating measurement systems with zero-chain traceability.

## Abstract (web summary)

Nanoionic memristive devices achieve stable quantum conductance at room temperature, enabling SI-traceable resistance standards and advancing self-calibrating electronics toward the implementation of national metrology institute services on chip.

## Main

Guaranteeing reliability and accuracy of measurements in all spheres of human knowledge is of ultimate priority to ensure the proper function of science and technology, and for the comfort and quality of our daily activities. To comply with this objective, national metrology institutes (NMIs) realize, develop and maintain primary standards of measurement units. The revision of the International System of Units (SI) in 2019^1,2^ represented a historic change of paradigm for metrology, opening a new perspective on the mise en pratique of the SI base units. Indeed, SI units are now mainly defined in terms of fundamental constants of nature defined by fixed numerical values. These fixed values are exact with zero uncertainty, and therefore no longer need to be measured. Accordingly, any experiment able to correlate measurable physical quantities to a fundamental constant, or a set of fundamental constants, fixed by the SI becomes a direct realization of the corresponding SI unit. This new paradigm is expected to revolutionize metrology as the science of measurement, making it possible to bring measurement technology and metrology out of NMIs directly to the end-user. In particular, the miniaturization and integration of NMI services on chip (for example, the NIST on a Chip programme^3^) working according to the principles of quantum physics can enable the realization of reliable SI-traceable self-referenced systems. In the field of electrical metrology, quantum phenomena such as the quantum Hall effect, the Josephson effect and single-electron transport effect have been widely explored for the practical realization of resistance, voltage and current electrical units, respectively^4^. Despite the recognized performances of metrological devices based on these quantum phenomena, the involved large size experimental set-ups and complexity of related measurements limit their realization almost exclusively to universities and metrology institutions. In the framework of the SI, the fundamental quantum of conductance, *G*_0_, is a quantity having a fixed numerical value with zero uncertainty (Supplementary Section 1). Hence, an experiment or device exhibiting a physical observable related to *G*_0_ can be exploited as a standard of resistance. Although it has been suggested that quantum effects in memristive devices could be exploited to overcome the main issues related to on chip integration of electrical standards^5^, an experimental verification is still missing. While several approaches have been followed to obtain quantum conductance levels in memristive devices^6–28^, their practical application has been hindered by a lack of substantial progress in programming and controlling such quantum levels.

In this Article, we report on a programmable resistance standard based on nanoionic memristive devices working in air, at room temperature, and implementable on chip. Besides introducing the electrochemical polishing effect to achieve reliable quantum conductance levels as multiples of *G*_0_, we provide a programming strategy that enables practical exploitation of quantum conductance effects even in presence of variability. Based on the results from an interlaboratory comparison involving three NMIs and three academic/research centres, we established consensus values of conductance states related to *G*_0_ and 2*G*_0_ that deviates from SI values by −3.8% and 0.6%, respectively. These results establish the basis for the realization of self-calibrating systems embedding intrinsic standards directly traceable to the SI.

## NMI services on chip

Metrological traceability is a fundamental requirement for any measurement process and is at the base of the intercomparability and validity of measurement outcomes, an essential need in science and in our daily activities. Traceability is defined as a property of a measurement result whereby the result can be related to a reference standard through a documented unbroken chain of calibrations, each calibration contributing to the overall measurement uncertainty^29^. In this framework, a chain of calibrations (or traceability chain) ensures the link between a common established reference (which underpins the comparability of measurement results) at the top of the chain and the measurement result given by an instrument on a lower step along the chain. Uncertainty increases after every step of the chain as the result of the contribution of the uncertainty associated with a new measurement that relates the instrument under calibration with the reference standard corresponding to that step.

**Fig. 1:**
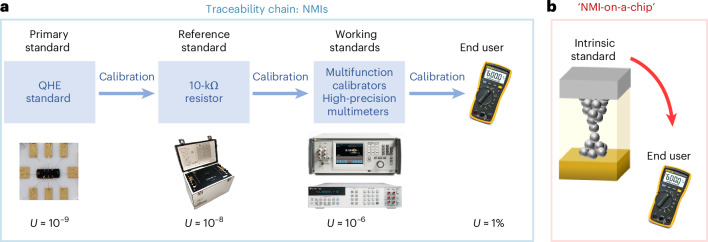
NMI on-a-chip. **a**, Conventional traceability chain for electrical resistance measurements. The chain starts with a comparison of the primary standard based on QHE with a first-level standard resistor (10 kΩ in the example given). In the second step of the traceability chain, this resistor standard is used to calibrate a second-order level of working standards as high-accuracy multifunction calibrators or digital multimeters. At the end of the chain, working standards are exploited to calibrate end-user equipment. Each step of the chain results in an increase of the measurement relative uncertainty *U*. The relative uncertainty *U* for the 10-kΩ example goes from some parts in 10^9^ (the uncertainty related to the limitations in the practical implementation of the quantum Hall resistance values) to some parts in 10^6^ (the typical specifications of precise multifunction calibrators or digital multimeters in the 10-kΩ range). At the end of the chain, we can find, for example, a hand-user multimeter with an accuracy specification of the order of 1%. **b**, Implementation of the memristive intrinsic standard of resistance directly in the end-user equipment, bringing NMI services on chip and allowing the realization of self-calibrating systems with zero-chain traceability.

Figure 1a shows an example of the conventional traceability chain for electrical resistance measurements. The reference standard for resistance based on the quantum Hall effect (QHE) (the standard having the highest metrological properties^30,31^) has been established for decades and is based on the von Klitzing constant *R*_K_ (ref.^32^). Notably, the potential use of the quantum anomalous Hall effect in metrology, which enables the realization of a resistance standard operating at zero external magnetic field, but still at cryogenic temperatures (∼35 mK) and vacuum conditions, has recently been demonstrated^33^. The traceability chain relies on a comparison of the primary standard based on the QHE with a first-level standard resistor that is then used to calibrate a second-order level of working standards such as multifunction calibrators or digital multimeters. Each step of the chain results in an increase of the measurement uncertainty *U* and, at the end of the chain, we can find, for example, a hand-user multimeter with a specification of the order of 1%. Each calibration in the traceability chain must be periodically repeated due to possible drifts of each measurement standard caused by operating time, environmental conditions and/or use. This means cost, long periods of unavailability of the measurement equipment and a lot of effort in the management process. Moreover, the QHE primary method has expensive and complex systems with highly demanding operating conditions because it need to operate in vacuum conditions, at very low temperatures (∼1 K) and under high applied magnetic fields (6–12 T) (Supplementary Section 2).

Memristive nanoionic devices, showing quantum conductance levels that are multiples of the fundamental quantum of conductance in air, at room temperature and implementable on chip, are therefore ideal candidates to be exploited as intrinsic standards of resistance. In this context, memristive devices make it possible to have an ‘NMI on chip’ available where the above-described traceability chain is no longer required (zero-chain traceability, Supplementary Section 3), enabling the realization of systems embedding the intrinsic standard that do not require any calibration (self-calibrating systems) (Fig. 1b).

## Electrochemical polishing in memristive devices

**Fig. 2:**
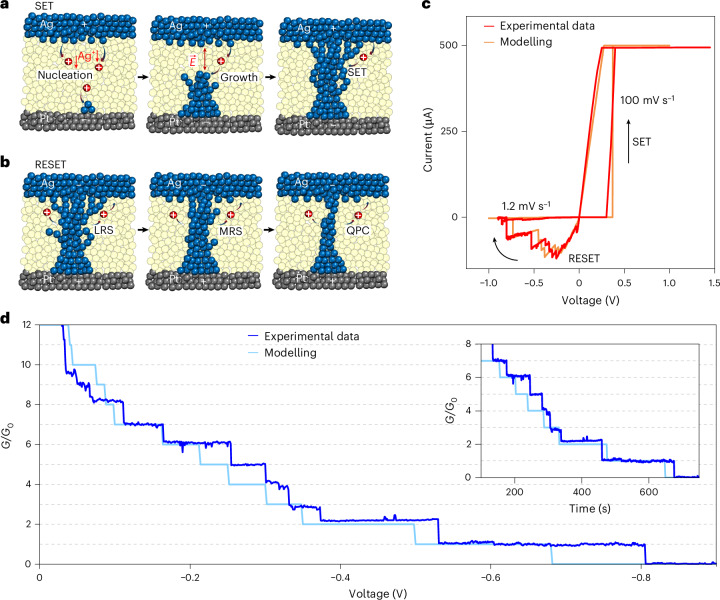
Quantum conductance levels stabilized by electrochemical polishing. **a**, Schematic representation of the SET process in a memristive cell, that is, an electrochemically driven process additionally accelerated by the electric field formed at the tip apex. The harsh conditions during filament growth in these nanoscale devices, characterized by extremely high current densities exceeding 10^6^ A cm^−2^ in conditions of high electric field acceleration (>10^8^ V cm^−1^), typically led to high unpredictability and variability of quantum conductance levels. **b**, The electrochemical polishing effect makes it possible to obtain more reliable quantum conductance levels by removing/dissolving first the unstable atoms at the contact configuration during the RESET process, but keeping the more stable ones. In this framework, the system evolves through discrete levels of conductance from a low-resistance state (LRS) to an intermediate metastable-resistance state (MRS) to a QPC. **c**, Example of a cycle showing abrupt SET obtained through a voltage sweep rate of 100 mV s^−1^ and RESET with discrete levels obtained by electrochemical polishing through a slow voltage sweep (1.2 mV s^−1^). **d**. The RESET process obtained through electrochemical polishing shows stable quantum conductance plateaus that are multiples of *G*_0_. Inset: the stability over time of the quantum conductance plateaus while sweeping the applied voltage.

Redox-based memristive devices are two-terminal nanoionics devices in which an ion-conductive (switching) film is sandwiched between two metal electrodes. The operation principle and functionalities of these cells rely on resistive switching effects related to the formation and rupture of a nanosized conductive filament within thin films (typically oxides or other chalcogenides) under the action of an electric field^34^. As a consequence of the progressive growth of the nanosized metallic whisker, quantum point contacts (QPCs) have been reported for short circuit conditions. The latter are typically achieved during the SET process (that is, the process that turns the device from an high-resistance state to a low-resistance state; Supplementary Section 4)^6,7,9^, which is electrochemically driven, but it is characterized by extremely high current densities and high electric fields (Fig. 2a). The electric field even increases during operation, due to the exponentially reducing distance between the tip apex and the electrode surface during the filament growth, until metallic contact is reached. Due to these extreme conditions, it is practically very difficult to control the filament growth process, resulting in large stochasticity in the shape, number of small dendrites/needles and the effective area of the formed contact. Accordingly, formation of QPCs is highly unpredictable and rather large filament(s) are formed under these harsh conditions. As a result, QPCs formed during the SET process largely vary in resistance value, and/or are highly unstable, even if formed at low voltages and currents. Alternatively, we propose forming QPCs during the RESET process (that is, the process that turns the device from a low-resistance state to a high-resistance state) in Ag/SiO_2_/Pt devices (Methods; details of materials and device configuration are given in Supplementary Section 5), exploiting the effect of electrochemical polishing (Supplementary Section 6). To obtain reliable QPC during RESET operation, after the SET process where a large filament is formed, we applied a sequence of voltages high enough to oxidize/dissolve the energetically unstable atoms in the filament and the peripheral nanoneedles in the contact configuration, but at the same time sufficiently low not to remove the more stable core atoms (Fig. 2b). Using this approach, despite Joule heating effects, we avoid high Faradaic currents and electric field acceleration of ionic reactions and transport, which ultimately results in more reproducible and reliable QPCs. During the partial RESET process, we neither break the contact, nor dissolve the filament entirely, but the filament is progressively narrowed, achieving *G* < 10*G*_0_, opposite to the abrupt SET process (Fig. 2c). In the partial RESET regime, the stepwise conductance changes correspond to integer multiples of *G*_0_ (Fig. 2d). Importantly, the quantum conductance states prepared by electrochemical polishing remain stable for several tens of seconds even under increasing negative voltage (Fig. 2d, inset), demonstrating a much higher stability and reliability compared with the QPC reached during the SET process. Thus, electrochemical polishing allows for predictable adjustment of stable quantum point levels, as required for the development of a quantum-based standard of resistance. A detailed discussion on the effect of electrochemical polishing in memristive devices can be found in Supplementary Section 7. It is worth remarking that the electrochemical polishing effect is best suited for memristive devices relying on the electrochemical metallization mechanism (ECM), as in our case (see Supplementary Section 8 for details).

Based on the electropolishing approach which aimed to slow RESET transitions, a stochastic model that considers conductance jumps ∼*G*_0_ accelerated by temperature (local power dissipation) and not by the electric field is proposed (Methods and Supplementary Section 9). This is related to the fact that redox dynamics and/or out-diffusion of metallic species that control the filament dissolution are sufficiently suppressed during the RESET process. As can be observed in Fig. 2c,d, the behavioural model results nicely track the experimental data (additional data in Supplementary Section 10).

## Interlaboratory comparison

**Fig. 3:**
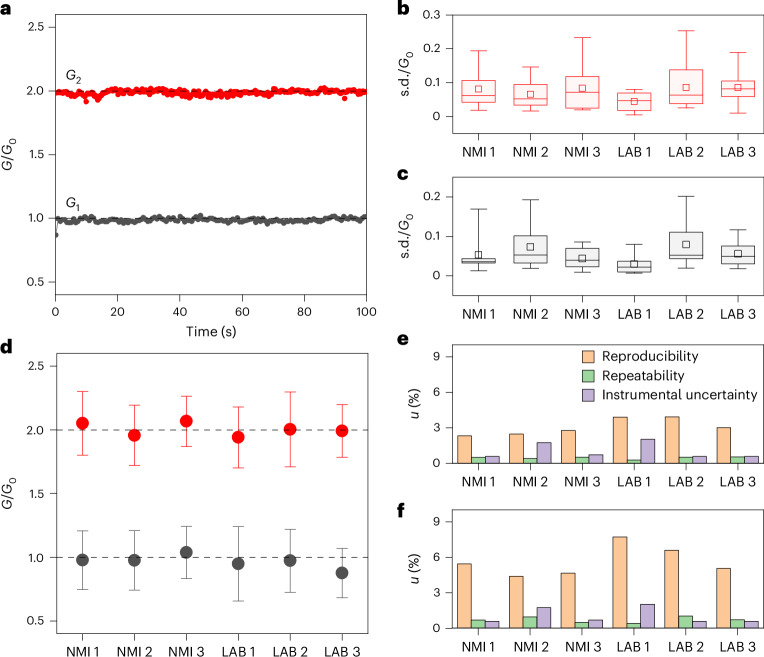
Quantum conductance levels and uncertainty budget. **a**, Stability of *G*_1_ and *G*_2_ quantum conductance levels achieved through a program-and-verify strategy in air and at room temperature. **b**,**c**, Repeatability of conductance values for *G*_2_ (**b**) and *G*_1_ (**c**), evaluated in terms of the s.d. over consecutive measurements of the programmed quantum levels, for the different NMIs and academic/research laboratories (LABs) participating in the interlaboratory comparison (see ‘Interlaboratory comparison’ in Methods for explanation of abbreviations). The sample size (*N*, the number of s.d. values for each participant) and the number of different devices used are presented in Supplementary Section 16. In box-plots, the midline represents the median of the *N* s.d. values, boxes show the 25th and 75th percentiles, and whiskers are the 5th and 90th percentiles. **d**, Reproducibility of conductance values for all participants in terms of the mean value of *N* mean values; the error bar represents the corresponding s.d. for *G*_1_ and *G*_2_. The sample size (*N*, the number of mean values) and the number of different devices used are presented in Supplementary Section 16. **e**,**f**, Relative standard uncertainty components of *G*_2_ (**e**) and *G*_1_ (**f**), related to reproducibility, repeatability and instrumental uncertainty for each participant.

**Extended Data Fig. 1:**
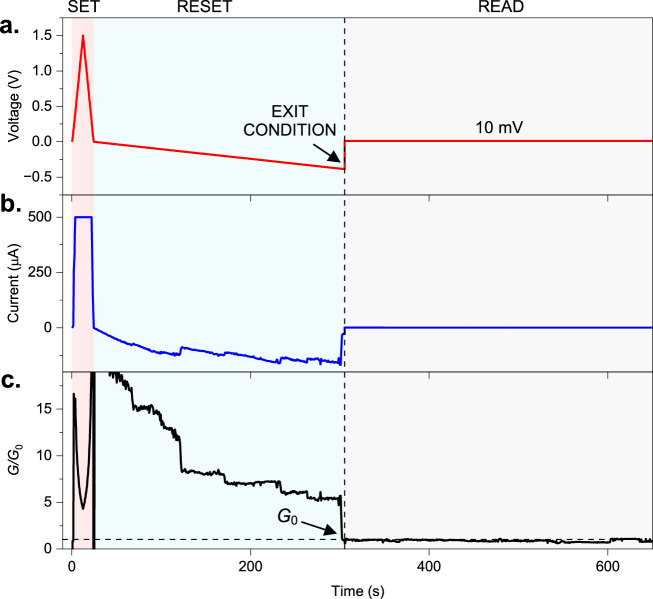
Program-and-verify approach to program the device to the desired quantum conductance levels. This approach consists in the following steps: *i)* SET of the device through a positive voltage sweep cycles in between 0 and 1.5 V (sweep rate of 96 mV s^−^^1^, compliance current of 500 µA externally controlled to avoid hard breakdown of the device) (red shadowed region); *ii)* RESET the device through a negative slow voltage sweep (2 mV s^−1^) from 0 down to -0.9 V to achieve a step-like decrease of device conductance due to quantum conductance effects (blue shadowed region); *iii)* READ of the desired quantum level with a continuous constant voltage, if the exit condition has been satisfied during the RESET step, the RESET cycle is interrupted and the control changes to a continuously positive read voltage of 10 mV (grey shadowed region). The exit condition is considered satisfied if the device exhibits during the time series at least 5 consecutive conductance measurements in the desired quantum level ± 0.5*G*_0_. Note that the device is continuously cycled until the desired quantum level has been obtained. **a**, Programming voltage; **b**, measured current; **c**, the device conductance.

**Extended Data Fig. 2:**
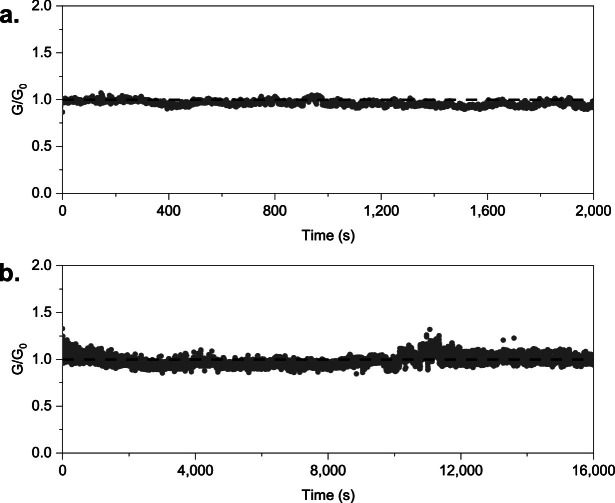
Stability of programmed quantum conductance levels. **a**. Example of a quantum conductance level measured at NMI 1 observed to be stable up to 2000 s under constant voltage reading of 10 mV (initial data are reported in Fig. 3a). **b**. Example of a quantum conductance level measured at NMI 3 observed to be stable up to 16000 s under constant reading voltage of 10 mV. Dashed lines represent the *G*_0_ reference value. Quantum conductance levels in panels a and b have been obtained through the program-and-verify approach, with negative voltage sweep for gradual RESET of 2 mV s^−1^ and 80 mV s^−1^, respectively.

An interlaboratory comparison study involving three NMIs and three academic/research laboratories was carried out with the purpose of measuring the achieved quantum reference values (see Methods for details). This activity included the establishment of a measurement protocol and data processing according to international standards and well-recognized best practices^35–37^. The measurement protocol was based on a ‘program-and-verify’ approach that aimed to generate and sustain the desired quantum conductance reference levels, making it possible to exploit memristive devices as a quantum standard of resistance while dealing with conductance fluctuations. This approach consists of selecting the desired discrete quantum conductance level during the gradual step-like RESET obtained through electrochemical polishing, and subsequent evaluation of the conductance level to be used as reference value by means of a constant applied voltage that, at the same time, sustains the conductance state (Extended Data Fig. 1). This applied read voltage is expected to stabilize the quantum conductance state by exerting quantum pressure forces due to recoil of flowing charge carriers that stabilize atomic-scale filaments and, thus, the quantum conductance level^38^. The program-and-verify approach reported here does not aim to fine-tune the device resistance as in conventional programming schemes for in-memory computing^39^, but rather to nudge the device towards the quantum conductance operational regime while checking if the desired quantum conductance level is achieved. Note also that a similar program-and-verify approach is conventionally exploited for the practical realization of a d.c. voltage standard based on the Josephson effect to appropriately select the desired voltage level to be used as the reference standard (Supplementary Section 11)^40^. Figure 3a reports examples of devices programmed in air and at room temperature through the program-and-verify approach at *G*_1_ and *G*_2_ conductance values related to the *G*_0_ and 2*G*_0_ quantum states, respectively. This program-and-verify approach makes it possible to achieve and sustain quantum conductance levels for up to 16,000 s (Extended Data Fig. 2). A comparison of the stability of quantum conductance values with previous works, including a comparison of device structures, switching mechanisms and programming approaches, is reported in Supplementary Section 12.

**Extended Data Fig. 3:**
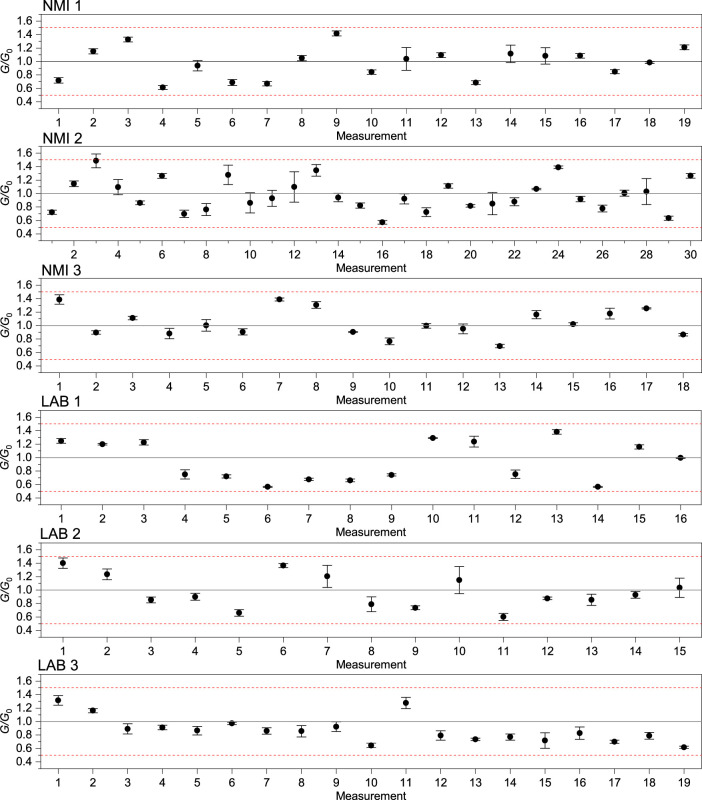
*G*_1_ conductance plateaus related to the *G*_0_ quantum level from the interlaboratory comparison. Each data point is the mean value and error bar is the standard deviation representing the repeatability evaluated over *n* (30 to 100) repeated measurements of the same conductance value. Red dashed lines represent boundaries of the exit condition of the program-and-verify approach, while grey line represents the *G*_0_ reference line.

**Extended Data Fig. 4:**
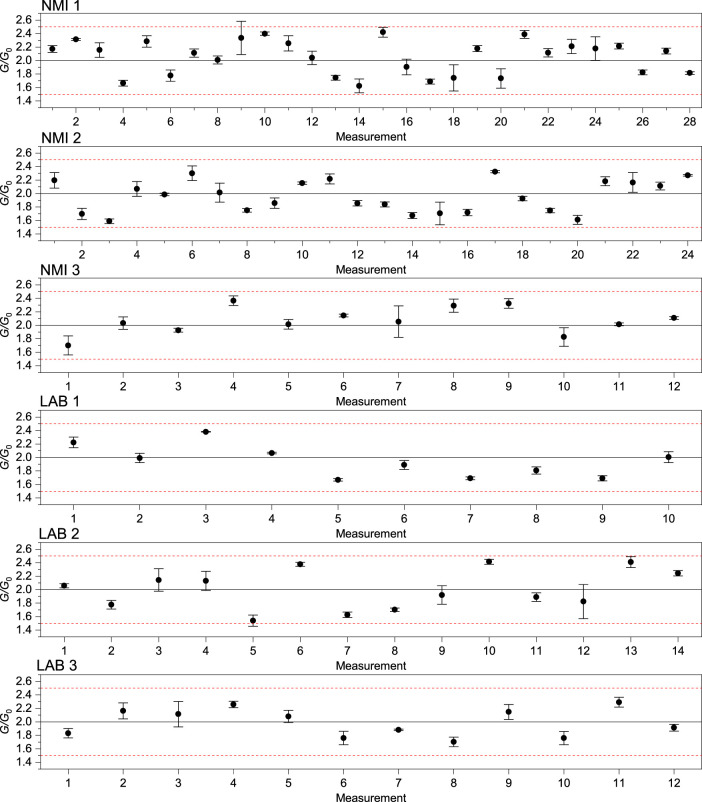
*G*_2_ conductance plateaus related to the 2*G*_0_ quantum level from the interlaboratory comparison. Each data point is the mean value and error bar is the standard deviation representing the repeatability evaluated over *n* (30 to 100) repeated measurements of the same conductance value. Red dashed lines represent boundaries of the exit condition of the program-and-verify approach, while grey line represents the 2*G*_0_ reference line.

For the interlaboratory comparison, each laboratory performed a series of programming levels, and for each programmed level a time series of conductance measurements was carried out. For each time series, a mean value of the measurement conductance was evaluated. The repeatability was defined as the s.d. of the time series values (Methods and Supplementary Section 13). The reproducibility of the conductance value was defined as the s.d. of the mean values corresponding to each programming level. The uncertainty related to the measurement equipment was extracted from the instrument specifications and measurement conditions (Methods and Supplementary Section 14). The repeatability, reproducibility and uncertainty of the experimental set-up all contribute to the overall uncertainty. The complete set of measurements is reported in Extended Data Figs. 3 and 4. The validation of the programming methodology is detailed in Supplementary Section 15.

**Extended Data Fig. 5:**
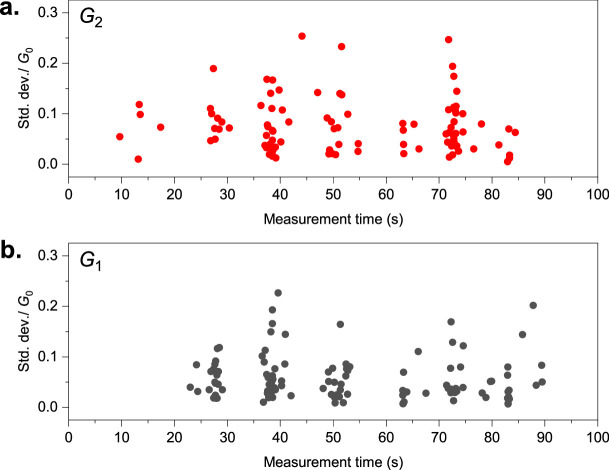
Measurement repeatability and time interval of consecutive measurements. Repeatability in terms of the standard deviation of *n* consecutive measurements evaluated for conductance levels related to 2*G*_0_ (panel **a**.) and to *G*_0_ (panel **b**.), for a measurement time up to 100 s. Each point has been evaluated by programming the device to a desired programmed quantum level and then evaluating the standard deviation of *n* (30 to 100) consecutive measurements of the conductance step. Graphs have been obtained through aggregated data analysis of results obtained by different labs in the interlaboratory comparison. In case of equal number of consecutive measurements, different measurement time intervals arise from different sample rates of measurement arising from different experimental equipment and their configuration parameters (for example NPLC – Number of Power Line Cycles, Filter, etc) exploited for the interlaboratory comparison. As can be observed, no significant trend can be observed in the standard deviation as a function of the measurement time interval, suggesting that the repeatability can be evaluated based on consecutive measurements performed on a small-time interval.

Figure 3b,c shows consistent repeatability in all laboratories in terms of the distribution of s.d. values for *G*_2_ and *G*_1_, respectively. No significant trends were observed in the s.d. as a function of the measurement time interval, showing that the contribution of repeatability to the measurement result can be evaluated based on consecutive measurements performed at relatively short time intervals, <100 s (Extended Data Fig. 5). This means that, for practical applications, it is possible to exploit the programmability of the memristive cell by on-demand programming it on the desired quantum conductance level when required without the need of long-term stability of the filament.

Figure 3d reports for each participant the mean value of the measured mean values and the corresponding s.d. of mean values of the considered quantum levels, and shows comparable reproducibility of programmed quantum levels in all the laboratories. As can be observed, the results for *G*_1_ and *G*_2_ are statistically consistent with expected *G*_0_ and 2*G*_0_ quantum values and no s.d. values can be observed among the laboratories, revealing that laboratory-to-laboratory variability can be considered negligible compared with the variabilities related to the reproducibility of quantum steps. In addition, it can be observed that cycle-to-cycle variability dominates over device-to-device variability (Supplementary Section 16). The cycle-to-cycle variability can be attributed to the peculiar dynamic trajectories of nanofilament reconfiguration leading to slightly different atomic configurations near the quantum point contact, resulting in slight variations of quantum conductance levels around integer multiples of *G*_0_, as analysed in a previous work^41^. Similarly, slight deviations from integer multiples of *G*_0_ were previously observed also in mechanically controllable break junctions^42–44^.

**Extended Data Fig. 6:**
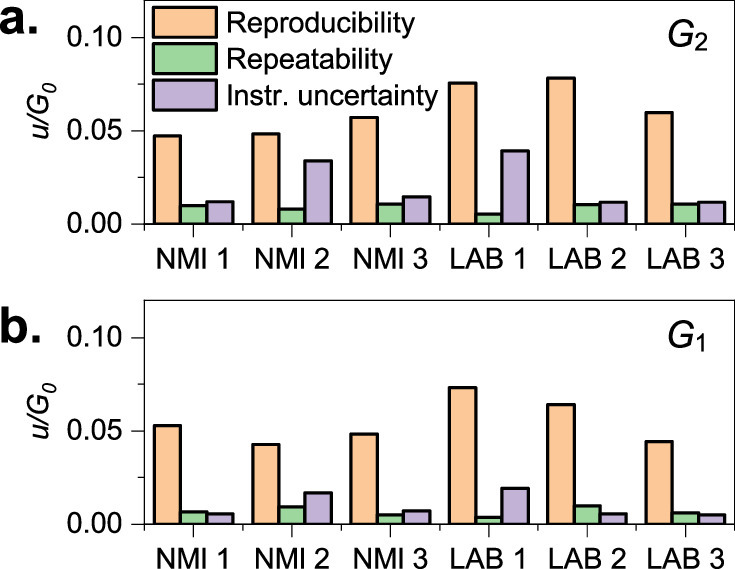
Uncertainty budget – absolute values. Absolute standard uncertainty components of *G*_2_ (panel a.) and *G*_1_ (panel b.) related to reproducibility, repeatability, and measurement equipment for each participant.

Figure 3e,f shows results for the estimation of the relative standard uncertainty components related to reproducibility, repeatability and measurement equipment of each partner for values of *G*_2_ and *G*_1_, respectively. The figure shows that the component of uncertainty related to reproducibility is dominant. In terms of percentage, higher reproducibility and repeatability can be observed when considering values of *G*_2_ with respect to *G*_1_ (Fig. 3e,f). However, statistical results show no significant discrepancies in terms of reproducibility and repeatability between the *G*_2_ and *G*_1_ conductance states when considering absolute uncertainty component values (Extended Data Fig. 6). Although the contribution from the uncertainty related to the measurement equipment is comparable among all laboratories, it is not negligible.

## Memristive devices as a standard of resistance

**Fig. 4:**
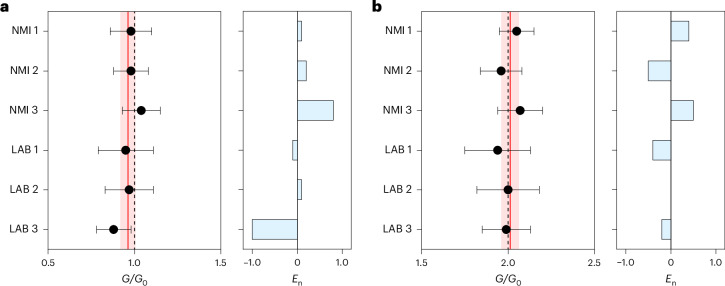
Resistance standard. **a**,**b**, Mean values of resistance standards based on memristive devices and corresponding expanded uncertainty (error bars) evaluated in different NMIs and laboratories (LABs) for *G*_1_ (**a**, left) and *G*_2_ (**b**, left). The red line is the consensus value *G*_cons_ of the participant’s results, the red shading is the corresponding expanded uncertainty *U*(*G*_cons_), and the black dashed line shows the *G*_0_ and 2*G*_0_ SI values. Right: the normalized error *E*_n_ of each participant exploited to qualify results with respect to the consensus value.

A consensus value *G*_cons_ and its corresponding expanded uncertainty *U*(*G*_cons_) were established based on the participant’s results for the investigated quantum conductance plateaus^36,37^. The aim of establishing a consensus value is to investigate a possible systematic deviation with respect to the exact quantization value. We choose as an estimate for *G*_cons_ the weighted mean of the values measured by participants^35^, while the combined uncertainty of the consensus value *U*(*G*_cons_) was estimated based on the participant’s combined uncertainties, which take into account all sources of uncertainties previously discussed and the coverage factor *k* calculated for a confidence level of 95% (see Methods for details). The normalized error *E*_n_ expresses the consistency of the results obtained by each participant with the consensus value^35,37^ (see Methods for details). Mean values of quantum levels with the corresponding expanded uncertainties for all laboratories and normalized error with respect to the consensus value for *G*_1_ and *G*_2_ are reported in Fig. 4a,b, respectively. The evaluation of |*E*_n_ | values (≤1.0 for all participants) shows that the measured conductance values belong to consistent datasets (Fig. 4a,b, right). *G*_cons_ was calculated to be (0.962 ± 0.043)*G*_0_ and (2.012 ± 0.051)*G*_0_ for *G*_1_ and *G*_2_, respectively. It turns out that the error of the consensus values for *G*_1_ and *G*_2_ is −3.8% and 0.6% with respect to the expected *G*_0_ and 2*G*_0_ SI values, respectively. In summary, consensus values agree with SI values because their deviation from SI values is well covered by the expanded measurement uncertainty.

## Conclusions

In this work, we demonstrated an intrinsic and programmable resistance standard, based on quantum physics, that, at the cost of higher uncertainty with respect to conventional standards based on QHE, operates in air, at room temperature and can be miniaturized down to the nanometre scale. This standard has the significant advantage of low operating voltages (of the order of 10 mV) and low operating currents (of the order of 1 µA) during reading operations that are ideally suited for practical applications. It is worth mentioning that, even if electrochemical processes underlying filament formation/rupture are affected by temperature^45^, electronic transport phenomena leading to quantum conductance levels arising at the QPC is theoretically not affected by temperature and, in principle, traceability of the physical observable is not affected when the device is operating at different temperatures (Supplementary Section 17). All these characteristics enable the realization of an intrinsic quantum standard of electrical resistance/conductance that can be made available at the lower levels of the presently adopted traceability chain and can be directly implementable on chip, opening new opportunities for metrological traceability of electrical quantities. This makes possible its incorporation into any type of electronic measurement instrumentation, including multimeters, resistance bridges, temperature bridges, voltage dividers, voltage amplifiers, current amplifiers, temperature controllers, reference resistors in analogue-to-digital converters, etc. By means of autocalibration and autoadjustment processes, the on-chip standard allows the realization of electrical equipment with zero-chain traceability. In this context, it must be acknowledged also that a standard of resistance coupled with a voltage standard can enable the practical realization of a current standard according to the Bureau International des Poids et Mesures.

At the present stage, the deviations of quantum conductance states of memristive devices with respect to SI values are higher than the accuracy required by primary metrology achieved in NMIs. Nevertheless, the availability of on-chip realization of the resistance standard can find applications (at lower levels of the traceability chain) where the periodical calibration of meters or sensors becomes inconvenient or impossible, and measurement traceability must be maintained over years or decades, in the spirit of the ‘NMI-on-a-chip’ paradigm. Examples lie in space applications, marine offshore environments, underground probes, sensing in remote locations, sensing in harsh environments and sensor networks.

For all these applications, several strategies can be exploited to improve the actual accuracy. From the device point-of view, further engineering of involved materials and stacked configurations combined with a refinement of the measurement protocol can be exploited to reduce the cycle-to-cycle variability, which is the dominant contribution to uncertainty. For example, the host matrix can be engineered to promote electrochemical dynamics leading to only selected atomic configurations closely related to the *G*_0_ SI value. Further strategies to reduce stochastic effects related to cycle-to-cycle variability, to improve the stability of the filament and to reduce conductance drifts over extended timescales include (1) optimizing the sweep rate during electrochemical polishing, (2) combining electrochemical polishing with a partial RESET process to reduce stochastic effects related to the formation of a completely new filament during each SET/RESET operation, and (3) electropolishing of the filament in a small voltage window after programming (here the strategy is to vary not only the total voltage range, but also components in the positive and negative voltage sign and the sweep rate) (Supplementary Section 7). Moreover, for practical applications, the accuracy can be improved by using *n* independent devices in parallel to produce an average value of quantum levels that could improve variability by an order of $\sqrt{n}$. Similarly, a viable strategy includes also the mitigation of variability by mediating among multiple realizations of the same desired quantum conductance value, by leveraging the device programmability.

While the on-chip implementation of a resistance standard based on memristive cells necessarily requires additional circuitry to drive and control its operations, the complexity and costs of this control circuit (which can be easily miniaturized through conventional complementary metal-oxide-semiconductor technology) is significantly lower than the complexity and costs associated with the maintenance of the traceability chain and routine calibrations based on the conventional QHE-based resistance standard. In this context, we envision that the practical implementation of the self-calibration concept can be based on the artefact calibration paradigm^46^, where an on-chip memristive cell coupled with a mixed-signal application-specific integrated circuit that takes care of cell programming/reading could replace the external reference standard. For all these purposes, the circuit design can exploit the here-described behavioural model of memristive devices working in the quantum regime which can be implemented in conventional circuit simulators.

## Methods

### Device fabrication

Memristive devices were fabricated by sandwiching a SiO_2_ insulator in between a platinum bottom electrode and a silver top electrode. The choice of silver as the top electrode is based on its electrochemical activity which allows dissolution of silver atoms and migration of Ag^+^ ions at lower voltages compared to other noble metals, while platinum was chosen as the counter-electrode because it is electrochemical inert (details on the selection of materials and device configuration can be found in Supplementary Section 5). The pad structure devices were fabricated on a thermally oxidized silicon substrate starting with the d.c. magnetron sputtering deposition (power, 200 W) of a TiO_2_ (10 nm) adhesion layer and a platinum (100 nm) bottom electrode. The homogeneous SiO_2_ film (20 nm) with a purity of 8N was deposited by radiofrequency (RF) magnetron sputtering with a sputtering power of 150 W in a processing gas mixture of 9 sccm argon and 1 sccm oxygen at 150 °C. Note that the choice of the 8 N SiO_2_ matrix is related to the very low level of impurities, a potential disturbing factor for achieving controlled conductance states. Also, the resulting SiO_2_ is rather stoichiometric and chemical and physical interactions with silver are not thermodynamically favourable. Following switching layer deposition, feature sizes of 50 × 50 μm^2^ were patterned by negative photolithography. Then, the Ag (20 nm) active top electrode was deposited by e-beam evaporation with a deposition rate of 0.01 nm s^−1^, followed by a d.c.-sputtered platinum (50 nm) capping layer. The role of the capping layer is to prevent degradation of the silver active electrode over time as required for long-term use of the device. A standard lift-off process was utilized for the final cleaning of devices, obtaining an Ag/SiO_2_/Pt cell with a top electrode size of 50 × 50 μm^2^.

### Device modelling

Departing from the experimental observation of well-defined conductance jumps and states, we model the RESET transition (the SET transition is also considered for generality) as a random generation of events related to the destruction of single quantum mode channels with conductance ∼*G*_0_. This is a stochastic version of a continuous behavioural compact model^47^ which has been successfully applied to memristors with different material systems, different switching modes (bipolar, unipolar, complementary and threshold switching) and for the SPICE simulation of neuromorphic circuits. The stochastic version of the model presented here was recently applied to valence change memory devices which show variability, but not quantum conductance jumps^48^.

The stochastic resistive switching model follows Chua’s approach^49^ to memristors and is based on two equations, one for the current and one for the internal memory variable. In our case, the memory state variable is the number of conducting channels, *n*_ch_, each of these channels contributing ∼*G*_0_ to the filament conductance. In a naive interpretation, each of these channels can be considered either as ‘atomic chains’ or as ‘quantized quantum transport modes’ in the filament constriction. This is a simple implementation of the Landauer theory for ballistic transport through an atomic-size constriction^50^. We consider that the SET/RESET transitions occur by successive discrete conductance jumps (events) corresponding to the creation/destruction of single conduction channels. For simplicity, we assume that each switching event increases or decreases the conductance by the same amount. However, this might not be completely realistic because several channels can be created/destroyed at the same time. During the RESET transition, we will consider that each jump is |Δ*G*| = *G*_0_. Given the experimental results, we impose that the first SET event is abrupt so that the device reaches the compliance limit in a single conductance jump. The creation/destruction of single channels will occur at random times during the application of the external electrical signal (voltage/current). For the sake of generality, we limit the number of channels to *n*_max_. This parameter is related to the maximum area of the filament created during electroforming. Under these conditions, the proposed memory equation is:

1 $$\frac{\text{d}{n}_{\mathrm{ch}}}{\text{d}t}=\frac{{n}_{\max }-{n}_{\mathrm{ch}}}{{\tau }_{{\rm{S}}}}-\frac{{n}_{\mathrm{ch}}}{{\tau }_{{\rm{R}}}}$$

where the two terms of the right-hand side (RHS) represent the SET and RESET transitions, and *τ*_S_ and *τ*_R_ are the SET and RESET characteristic times, respectively. Because the SET transition resembles the dielectric breakdown process and is strongly accelerated by the electric field, an exponential voltage dependence for *τ*_S_ is assumed:

2 $${\tau }_{{\rm{S}}}(V\,)={\tau }_{{\rm{S}}0}\exp \left[-{\gamma }_{{\rm{S}}}(V-I{R}_{{\rm{S}}})\right]$$

where *γ*_S_ is the acceleration factor, *τ*_S0_ is the time scale prefactor, *I* is current, *V* is voltage and *R*_S_ is the series resistance. On the other hand, consistently with the electropolishing interpretation, the RESET transition is assumed to be controlled by the oxidation/reduction dynamics and/or by the out-diffusion of species to the filament surroundings. Because both processes are strongly accelerated by temperature, we neglect voltage acceleration (as discussed within the electropolishing interpretation) and we only consider the local temperature rise related to the power dissipated in the filament, $P=I\left(V-I{R}_{{\rm{S}}}\right)$. Assuming an Arrhenius temperature dependence as a first-order approximation, the characteristic RESET time, *τ*_R_, can be described as:

3 $${\tau }_{{\rm{R}}}\left(V\,\right)={\tau }_{{\rm{R}}0}\exp \left[\frac{{E}_{{\rm{a}}}}{{K}_{{\rm{B}}}(T+{R}_{\mathrm{TH}}P)}\right]$$

where *τ*_R0_ is the RESET scale prefactor, *E*_a_ is the activation energy, *K*_B_ is the Boltzman constant, *T* is the external temperature and *R*_TH_ is the thermal resistance. The thermal resistance has been described in the literature in terms of two parallel paths for heat evacuation^51^. The longitudinal thermal resistance, *R*_L_, corresponding to heat transport along the channel (related to the electrical conductivity) and the transverse resistance, *R*_T_, associated with heat transport towards the surrounding material. The latter is independent of the filament size to the first order, while *R*_L_ is inversely proportional to the filament area, represented here by *n*_ch_, which is proportional to the area. Thus, we can write ${R}_{{\rm{L}}}={K}_{{\rm{L}}}/{n}_{\mathrm{ch}}$, where *K*_L_ is a constant. The total thermal resistance is given by the parallel combination of *R*_L_ and *R*_T_, so that ${R}_{\mathrm{TH}}=\left({K}_{{\rm{L}}}{R}_{{\rm{T}}}\right)/\left({n}_{\mathrm{ch}}{R}_{{\rm{T}}}+{K}_{{\rm{L}}}\right)$. It is worth remarking that we included only description of thermal dissipation with a phenomenological approach based on macroscopic parameters such as thermal resistances. While in principle quantum thermal effects cannot be ruled out, experimental works pointed out that these effects only become not negligible in the low-temperature regime^52^, that is, far away from the room temperature conditions of our work.

Because *τ*_S_ has a strong exponential dependence on voltage, it emerges that ${\tau }_{{\rm{S}}}\ll {\tau }_{{\rm{R}}}$ for positive voltages and ${\tau }_{{\rm{S}}}\gg {\tau }_{{\rm{R}}}$ for negative voltages. Because of this, we can separately consider the SET and RESET transitions with two separate differential equations. One for the SET:

4 $$\frac{{\rm{d}}{n}_{\mathrm{ch}}}{{\rm{d}}t}=\frac{{n}_{\max }-{n}_{\mathrm{ch}}}{{\tau }_{{\rm{S}}}}$$

And one for the RESET:

5 $$\frac{{\rm{d}}{n}_{\mathrm{ch}}}{{\rm{d}}t}=-\frac{{n}_{\mathrm{ch}}}{{\tau }_{{\rm{R}}}}$$

As far as the current is concerned, we have considered:

6 $$I\left(V\,\right)=\frac{{n}_{\mathrm{ch}}{G}_{0}}{1+{n}_{\mathrm{ch}}{G}_{0}{R}_{{\rm{S}}}}V+{I}_{{\rm{B}}}\,\sinh \left[\eta \left(V-I{R}_{{\rm{S}}}\right)\right]$$

where *η* is a shape parameter related to the potential barrier at the constriction when there are no conducting channels. The first term corresponds to the conduction through the *n*_ch_ channels, and the second to the background tunnelling regime, that is, when the filament has a gap. Although the considered voltage dependence of the background current can be discussed, this is not relevant to our work because we focus on situations where there is at least one conducting channel with a conductance which is generally much larger than that of the background. Finally, notice that *n*_ch_ couples the current and memory equations.

For the generation of random events, we follow an ‘on-the-fly’ method. If the number of events (conductance jumps) is *n*(*t*), the event generation rate is $\lambda \left(t\right)={\rm{d}}n\left(t\right)/{\rm{d}}t$. During the SET transition, ${n}_{\mathrm{ch}}=n\left(t\right)$ so that $\lambda \left(t\right)={\rm{d}}{n}_{\mathrm{ch}}/{\rm{d}}t$, while during RESET ${n}_{\mathrm{ch}}={n}_{\max }-n\left(t\right)$, so that $\lambda \left(t\right)=-{\rm{d}}{n}_{\mathrm{ch}}/{\rm{d}}t$. Thus, the event generation rates can be obtained from equations (4) and (5) so that ${\lambda }_{{\rm{S}}}={(n}_{\max }-{n}_{\mathrm{ch}})/{\tau }_{{\rm{S}}}$ and ${\lambda }_{{\rm{R}}}={n}_{\mathrm{ch}}/{\tau }_{{\rm{R}}}$ during SET and RESET, respectively. Since ${n}_{\max } > {n}_{\mathrm{ch}}$ at any time, both generation rates are always positive as they must be. For the RESET transition, we will depart from an initial number of channels, *n*_init_, which are the ones generated during the previous SET transition.

The events are generated with a random number *u* uniformly distributed between 0 and 1 along the simulation time. The simulation time is discretized in steps Δ*t* which are small enough so that *λ*(t) can be assumed to be constant during Δ*t*. It can be shown that under these conditions, the random time to a subsequent event at time *t* is $\Delta {t}_{{\rm{u}}}=-\mathrm{ln}(u)/\lambda (t)$. During the simulation, if $\Delta {t}_{{\rm{u}}} < \Delta t$ an event is generated at time *t*, otherwise, the event is rejected. Details on modelling are discussed in Supplementary Section 9.

### Interlaboratory comparison

An interlaboratory comparison involving six participants was carried out for the electrical characterization of quantum conductance levels in memristive devices, with the aim of testing the intrinsic standard of electrical conductance (or resistance) and for evaluating laboratory-to-laboratory variability. For this purpose, samples assumed to be identical were distributed among participants and a common measurement protocol was defined. The participants were the following institutions: Istituto Nazionale di Ricerca Metrologica (Italian Institute of Metrology, NMI 1), Instituto Português da Qualidade (Portuguese Institute of Metrology, NMI 2), Turkiye Bilimsel ve Teknolojik Arastirma Kurumu (Turkish Institute of Metrology, NMI 3), Forschungszentrum Juelich GmbH (LAB 1), Fundación IMDEA Nanociencia (LAB 2) and Politecnico di Torino (LAB 3).

### Measurement protocol

The equivalence of the measurements across the different laboratories was ensured by establishing and agreeing a measurement protocol that defines standardized measurement conditions to program, accept and stabilize the quantum conductance level, and defines the methodology to measure its conductance value under steady conditions (an example of the device programming methodology is reported in Extended Data Fig. 1). The generation of the quantum conductance states is achieved by running sequential SET/RESET cycles where an applied voltage to the two terminals of the device is swept between +1.5 V and −0.9 V. The positive part of the sweep (SET cycle) has a sweep rate of 96 mV s^−1^ (voltage steps of 50 mV). The negative sweep (RESET cycle) has a slower sweep rate of 2 mV s^−1^ (voltage steps of 1 mV). The current compliance was established as 500 µA and 10 mA for the positive and negative cycles, respectively. The voltage at the terminals of the device and the current that flows through it are continuously measured over SET/RESET cycles, and the corresponding conductance state is obtained for each applied voltage step. The formation of the quantum conductance steps during the RESET is continuously verified and a criterion to detect and accept *G*_1_ and *G*_2_ conductance states related to *G*_0_ and 2*G*_0_ quantum values, respectively, was established. If the last five consecutive measurements of the conductance state lay within either *G*_0_ ± 0.5*G*_0_ or 2*G*_0_ ± 0.5*G*_0_ (censoring interval), the sweep RESET cycle is interrupted, and a continuous read voltage of 10 mV is applied. The measurement of the step conductance value starts under this fixed applied control voltage and continues as long as it remains in the intervals [0.5*G*_0_; 1.5*G*_0_] or [1.5*G*_0_; 2.5*G*_0_]. The measurements were made at room temperature and under normal environmental conditions. The equipment used was a source meter (different equipment was used by the participants, as detailed in Supplementary Section 14) in autorange mode. The above-described methodology makes it possible to deal with the stochasticity of the conductive filament formation process establishing an initial limit to the variability around the nominal values of the desired quantum conductance steps (the validation of this programming methodology is discussed in Supplementary Section 15). Note that all measurements not strictly following the established comparison protocol were not considered for the interlaboratory comparison.

### Evaluation of results and uncertainty budget

The evaluation of the average value and the variability of the programmed quantum steps was made from the observation of the measurements taken under repeatability and reproducibility conditions (described in appendix 2 of ref. ^4^). Here, repeatability conditions are understood as measurements of a specific device taken consecutively, while reproducibility is considered as the variability of the measurements taken from cycle-to-cycle operation of a specific device and programmed state as well as from device-to-device operations.

For each participant, *j*, the arithmetic mean and the experimental standard deviation were calculated for each series *i* of *n*_*i*_ values:

7 $${\bar{G}}_{j,i}=\frac{1}{{n}_{i}}\mathop{\sum }\limits_{a=1}^{{n}_{i}}{G}_{i,a}$$

8 $${s}_{j,i}=\sqrt{\frac{1}{{n}_{i}-1}\mathop{\sum }\limits_{a=1}^{{n}_{i}}{\left({G}_{i,a}-{\bar{G}}_{j,i}\right)}^{2}}$$

The s.d. given by equation (8) is an estimate of the repeatability^53,54^ associated with series *i* of a programmed quantum conductance state measured by participant *j*. Only series with a minimum of 30 consecutive values and limited to a maximum of 100 values were considered as a fixed condition in this data evaluation (Supplementary Section 13). As each participant measured *N*_*j*_ series and there are series with different numbers of values, a polled standard deviation^55^ ${s}_{{\rm{p}},\,j}^{2}$ is calculated based on the following equation for its variance:

9 $${s}_{{\rm{p}},\,j}^{2}=\frac{{\sum }_{i=1}^{{N}_{j}}\left({n}_{i}-1\right)\times {s}_{j,i}^{2}}{{\sum }_{i=1}^{{N}_{j}}\left({n}_{i}-1\right)}$$

${s}_{{\rm{p}},\,\,j}$ is therefore a weighted average of the ${N}_{j}$ s.d. where the number of degrees of freedom $\left({n}_{i}-1\right)$ is the weight of each series.

For each participant, an average of the mean values obtained from the ${N}_{j}$ series and the experimental s.d. is calculated as:

10 $${\bar{\bar{G}}}_{j}=\frac{1}{{N}_{j}}\mathop{\sum }\limits_{i=1}^{{N}_{j}}{\bar{G}}_{j,i}$$

11 $${S}_{j}=\sqrt{\frac{1}{{N}_{i}-1}\mathop{\sum }\limits_{i=1}^{{N}_{j}}{\left({\bar{G}}_{j,i}-{\bar{\bar{G}}}_{j}\right)}^{2}}$$

The evaluation of the reproducibility of the programmed quantum conductance steps was based on the s.d.^53,54^ given by equation (11). Because the values obtained by each participant for each step are from different cycles and different devices, the reproducibility obtained is the result of cycle-to-cycle and device-to-device variability.

The measurement of quantum conductance states associated with each participant is expressed by the following measurement equation:

12 $${{G}_{j}={\overline{\bar{G}}}_{j}+{S}_{j}+{s}_{{\rm{p}},\,j}+e}_{j}$$

where ${\bar{\bar{G}}}_{j}$ is the mean value calculated by participant *j*, *S*_*j*_ is the related experimental s.d. according to equations (10) and (11), *s*_p, *j*_ is the repeatability of the measurements according to equation (9), and *e*_*j*_ is the error related to the accuracy of the measurement equipment used. It is assumed that these input variables are statistically random variables where *S*_*j*_, *s*_p, *j*_ and *e*_*j*_ have an expectation value equal to zero and a s.d. estimated based on the experimental values presented before (*S*_*j*_ and *s*_p, *j*_) and in the manufacturing specifications of the equipment used (for *e*_*j*_). Note that random effects, including cycle-to-cycle variability but also variations related to small variations in the room temperature, humidity levels or even small fluctuations from the measurement set-up, are included in the estimation of the uncertainty component of the quantities *S*_*j*_ and *s*_p, *j*_, even if each specific contribution has not been disentangled.

The measuring uncertainty of *G*_*j*_ can be estimated by applying the law of propagation of uncertainties^55^ to equation (12):

13 $${u}^{2}\left({G}_{j}\right)={u}^{2}\left({S}_{j}\right)+{u}^{2}\left({s}_{{\rm{p}},\,j}\right)+{u}^{2}\left({e}_{j}\right)$$

where ${u}^{2}\left(x\right)$ is the variance (square of standard uncertainty) associated with the variable *x* and *u*^2^(*G*_*j*_) is the square of the combined uncertainty of *G*_*j*_.

The standard uncertainties of *S*_*j*_ and *s*_p, *j*_ are estimated by the corresponding s.d. of the mean:

14 $$u\left({S}_{j}\right)=\frac{1}{\sqrt{{N}_{j}}}{S}_{j}$$

15 $$u\left({s}_{{\rm{p}},\,j}\right)=\frac{1}{\sqrt{{\sum }_{i=1}^{{N}_{j}}\left({n}_{i}\right)/{N}_{j}}}{s}_{{\rm{p}},\,j}$$

The relative standard uncertainty of *e*_*j*_ is calculated from the combined relative uncertainty of the measurement of the voltage, ${u}_{{\rm{r}}}^{2}\left(U\right)$, and current, ${u}_{{\rm{r}}}^{2}\left(I\right)$:

16 $${u}_{{\rm{r}}}\left(e\right)=\sqrt{{u}_{{\rm{r}}}^{2}\left(U\,\right)+{u}_{{\rm{r}}}^{2}\left(I\,\right)}$$

The relative uncertainties of the measured voltage *U* and current *I* are estimated assuming a rectangular probability distribution for the voltage and the current measuring error with the plus/minus limits given by the manufacturing specifications of the equipment, usually identified as ‘accuracy’ (Supplementary Section 14):

17 $${u}_{{\rm{r}}}\left(U\,\right)=\frac{1}{\sqrt{3}}\frac{{U}_{\mathrm{accuracy}}}{U}$$

18 $${u}_{{\rm{r}}}\left(I\,\right)=\frac{1}{\sqrt{3}}\frac{{I}_{\mathrm{accuracy}}}{I}$$

Following the international recommendation to express the final measuring uncertainty with a coverage probability of approximately 95%^56,57^, the expanded uncertainty *U*(*G*_*j*_) is calculated following the equation:

19 $$U\left({G}_{j}\right)=k\times u\left({G}_{j}\right)$$

where *k* is the coverage factor calculated according to annex G of ref. ^55^.

### Evaluation of consensus value

The evaluation of the results achieved by the participants was done by comparing individual results with a consensus value^36,37^. The consensus value is established based on all results from the participants^37^, using a weighted average of their values^35^:

20 $${G}_{\mathrm{cons}}=\left(\sum _{\,j=1}^{6}{w}_{j}\times {G}_{j}\right)\left/\left(\sum _{\,j=1}^{6}{w}_{j}\right)\right.$$

where the weighting factors are given by:

21 $${w}_{j}=1/{u}^{2}\left({G}_{j}\right)$$

The combined uncertainty of the consensus value is estimated based on the participant uncertainties as follows:

22 $$u\left({G}_{\mathrm{cons}}\right)=\sqrt{1\left/\sum _{j=1}^{6}\right.{w}_{j}}$$

And the related expanded uncertainty is given assuming a coverage factor *k* = 2 (ref. ^35^):

23 $$U\left({G}_{\mathrm{cons}}\right)=2\times u\left({G}_{\mathrm{cons}}\right)$$

To identify an overall consistency of the results produced by this approach, a chi-square test was applied to the input values^35^:

24 $${\chi }_{\mathrm{obs}}^{2}=\mathop{\sum }\limits_{j=1}^{n}\left[{\left({G}_{j}-{G}_{\mathrm{cons}}\right)}^{2}/{u}^{2}\left({G}_{j}\right)\right]$$

The result of the test is considered to fail if $\Pr \left\{{\chi }^{2}\left(\nu \right) > {\chi }_{\mathrm{obs}}^{2}\right\} < 0.05$ where Pr is the ‘probability of’, ${{\chi }}^{2}\left(\nu \right)$ is the expected theoretical value of a chi-squared distribution for $\nu$, and $\nu$ is the degrees of freedom, which is the number of input values *n* minus 1 (in this case, 5). If the consistency check does not fail, then *G*_cons_ can be accepted as the consensus value and *U*(*G*_cons_) can be accepted as its expanded uncertainty. Values obtained for the interlaboratory comparison were ${\chi }_{\mathrm{obs}}^{2}=6.3$ and ${{\chi }}^{2}\left(5\right)=11.1$. As ${\chi }_{\mathrm{obs}}^{2}\le {\chi }^{2}\left(5;0.05\right)$, the consistency of the participant’s values and the calculated consensus value was demonstrated, thus the obtained *G*_cons_ is the consensus value and *U*(*G*_cons_) is its expanded uncertainty.

To qualify the result of each participant related to the consensus value, the normalized error^35,37^, *E*_n, *j*_, was calculated by:

25 $${E}_{{\rm{n}},\,j}=\left({G}_{j}-{G}_{\mathrm{cons}}\right)/\sqrt{{U}^{2}\left({G}_{j}\right){-U}^{2}\left({G}_{\mathrm{cons}}\right)}$$

The value of *E*_n, *j*_ has the following meaning: if |*E*_n, *j*_ | ≤ 1.0, the result is consistent (passed); if |*E*_n, *j*_ | > 1.0, the result is inconsistent (failed). For all participants, results were observed to be consistent with the established consensus value. Based on statistical analysis, higher values of |*E*_n, *j*_ | (even if always ≤1.0) cannot be ascribed to eventual systematic errors affecting the measurement that are not being adequately corrected or considered in the evaluation of measurement uncertainty.

## Online content

Any methods, additional references, Nature Portfolio reporting summaries, source data, extended data, supplementary information, acknowledgements, peer review information; details of author contributions and competing interests; and statements of data and code availability are available at 10.1038/s41565-025-02037-5.

## Supplementary information

Supplementary Information Supplementary Sections 1–17.

## Data Availability

The data that support the findings of this study are available via Zenodo at 10.5281/zenodo.16788655 (ref. ^58^). All other data are available from the corresponding authors.
