# Supplementary material for: A quantum resistance memristor for an intrinsically traceable International System of Units standard
Source: Nat Nanotechnol. 2025 Oct 27;20(12):1884–90. doi: 10.1038/s41565-025-02037-5 (PMC12727501; doi:10.1038/s41565-025-02037-5)
Supplement: Supplementary file 1 — Supplementary Sections 1–17. [file 41565_2025_2037_MOESM1_ESM.pdf]

nature nanotechnology

Supplementary information https://doi.org/10.1038/s41565-025-02037-5

A quantum resistance memristor for an intrinsically traceable International System of Units standard

In the format provided by the authors and unedited

A quantum resistance memristor for an intrinsically traceable International System of Units standard

Gianluca Milano*, Xin Zheng, Fabio Michieletti, Giuseppe Leonetti, Gabriel Caballero, Ilker Oztoprak, Luca Boarino, Özgür Bozat, Luca Callegaro, Natascia De Leo, Isabel Godinho, Daniel Granados, Itir Koymen, Mariela Menghini, Enrique Miranda, Luís Ribeiro, Carlo Ricciardi, Jordi Suñe, Vitor Cabral*, Ilia Valov*

Supplementary Information

Supplementary Section 1: The fundamental quantum of conductance G_0

Supplementary Section 2: Conventional resistance standard based on Quantum Hall Effect

Supplementary Section 3: The concept of zero-chain traceability

Supplementary Section 4: Literature survey on programming memristive devices in the quantum regime

Supplementary Section 5: Details on the selection of materials and device configuration

Supplementary Section 6: Working principles of electrochemical polishing

Supplementary Section 7: Electrochemical polishing effect in memristive devices

Supplementary Section 8: On the electrochemical polishing effect in ECM and VCM cells

Supplementary Section 9: Details on the modeling approach

Supplementary Section 10: Experimental and modeling RESET characteristics

Supplementary Section 11: "Program and verify" approach for practical realization of a voltage standard based on Josephson effect

Supplementary Section 12: On the stability of quantum conductance values

Supplementary Section 13: Evaluation of repeatability of the quantum conductance value

Supplementary Section 14: Specification of equipment and corresponding measurement accuracy

Supplementary Section 15: Statistical validation of the programming methodology

Supplementary Section 16: Cycle-to-cycle and device-to-device variability

Supplementary Section 17: On the effect of temperature on quantum conductance levels

1. The fundamental quantum of conductance G_0

The quantum of electrical conductance $G_0 = 2e^2/h$, and the von Klitzing constant $R_K = 2\,(G_0)^{-1} = h/e^2$ are both simple arithmetic combinations of two fundamental constants of nature, the elementary electron charge e and the Planck constant h. Since 2019, "The International System of Units, the SI, is the system of units in which [...] the Planck constant, h, is $6.62607015 \times 10^{-34}$ J s; [...], the elementary charge, e, is $1.602176634 \times 10^{-19}$ C [...]. The numerical values of the seven defining constants have no uncertainty." [SI Brochure[1], Sec. 2.2., p. 127]. Therefore, in the SI both $G_0 = 7.748091729... \times 10^{-5}$ S, and $R_K = 2\,(G_0)^{-1} = 25812.80745...$ Ω have fixed values, with no uncertainty. The dots (...) represent further digits that can be calculated to any degree of precision required.

2. Conventional resistance standard based on Quantum Hall Effect

An example of the experimental setup required for the practical realization of the conventional resistance standard is reported in Supplementary Figure 1a. Here, a low temperature measurement of the Quantum Hall Effect (QHE) device is performed by means of a cryostat that, connected to the vacuum system, can reach a temperature of ~1 K by liquid helium (^{4}He). A superconducting magnet placed inside the cryostat is exploited to generate the required magnetic field (e.g., 6 T to 12 T) for operating QHE devices. Associated instrumentation to this system includes a variable current source to control the value of the magnetic field, temperature sensors, low-pressure sensors, voltmeter to measure the Hall voltage, V_{H} and horizontal Voltage, V_{xx} high stable current source to drain the Quantum Hall resistance sample. Costs associated to a typical ^{4}He system relies in the amount of liquid helium (≈ 200 L per one week of operation) each time it is operated and the needed time in its preparation (cooling down from room temperature to the operating temperature) and routine test verification to assure that the required conditions of minimization/elimination of error sources are fulfilled.

An example of QHE device based on a quantum heterostructure of GaAs/AlGaAs and the equivalent circuit representation are reported in Supplementary Figure 1b and c, respectively. Here, the working principle is based on the formation of a two-dimensional electron gas (2DEG) at the interface between GaAs and AlGaAs that leads to quantum confinement effects, with the formation of a set of discrete energy levels.[2] As a consequence, when the device is driven by a constant current I, the quantum Hall effect is observed when the Hall voltage V_{H} is constant for an interval of the applied value of the magnetic field B and the horizontal voltage V_{xx} has values very close to zero.

The level of the expanded uncertainty reached with this primary system is in the order of some parts in 10^9 as has been demonstrated in the level of agreement of these systems resulting from on-site bilateral comparisons performed between BIPM (Bureau International des Poids et Mesures) and different National Metrology Institutes around the World.[3]

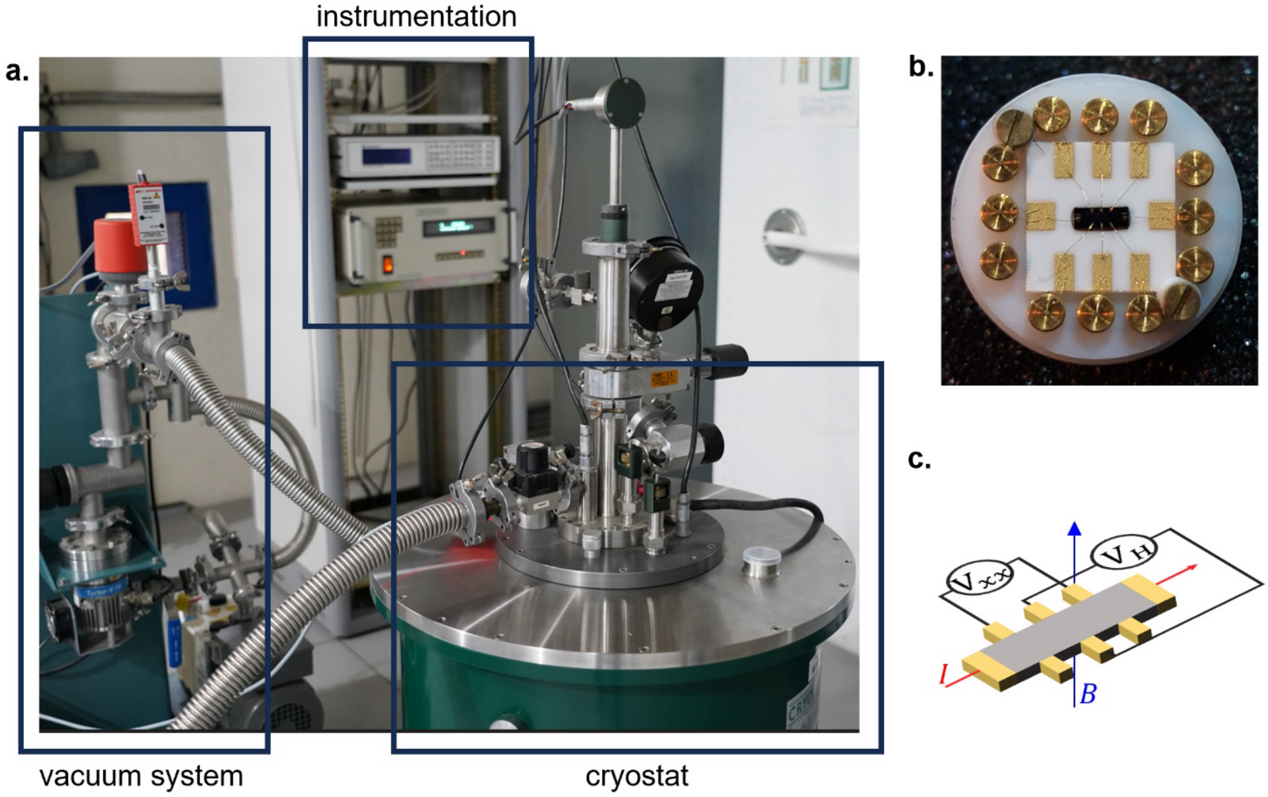

Supplementary Figure 1 | Experimental setup required for the conventional resistance standard based on QHE. a. Experimental setup at the Portuguese National Metrological Institute (Instituto Português da Qualidade) exploited for the practical realization of the conventional QHE resistance standard, **b.** Image of the QHE device based on a GaAs/AlGaAs heterostructure, and **c.** the equivalent circuit representation.

3. The concept of zero-chain traceability

The concept of *zero-chain traceability* was made popular by the National Institute of Standards and Technology in launching their "NIST-on-a-chip" program[4] of intrinsically accurate, quantum-based measurement systems and devices available to the final user without the need of calibration from National Metrology Institutes. The European Metrology Network for Quantum Technologies (EMN-Q) strategic research agenda[5] places zero-chain traceability as a major target of European quantum metrology development, highlights the benefits, for calibration laboratories and final users, of "[...] novel 'intrinsically referenced' calibration systems, offering direct SI traceability to primary quantum standards [...] towards the concept of *zero-chain traceability*, which is no longer reliant on traceability provided by or through NMIs."

4. Literature survey on programming memristive devices in the quantum regime

Quantum conductance has been observed in air at room temperature in both SET[6–12] and RESET[6–8,11,13] operations. The specific methods reported to obtain controllable quantum conductance levels and corresponding stability of quantum conductance levels can be summarized as follows:

Slow voltage sweep mode;[9–11,13–16] A suitable low voltage sweep rate (≈ mV·s^{-1}) is applied to slow the growth of the filament and to observe quantum conductance plateaus in SET process.[9,10,14,15] The quantum conductance < 10 G_0 obtained by this method has a stability of some tens to thousands of seconds, but the retention failure comes earlier with decreased conductance.[15] Some works also reported conductance quantization during RESET process with slow voltage sweep mode,[8,11,13] but there is still no detailed study about the stability and reproducibility of quantum conductance levels obtained in this operational regime. Banerjee and Hwang[17] demonstrated an electrically controllable break junction (ECBJ) with a structure Cu/Ti/HfO_2/TiN, which revealed a controllable conductance at high temperature. However, such a type of cell is expected to be more sensitive to oxygen and humidity[18,19] compared to the here reported SiO_2 based ECM cell with a pure metal Ag filament.

Current-controlled SET process;[20–25] By controlling the current under low current sweep rate[20–22] or with a different current compliance[23,24], multiple conductance levels were observed in SET process. The quantum conductance < 10 G_0 obtained by this method has a stability of some seconds to thousands of seconds,[20,24] but the retention failure accelerates with decreased conductance[24]. It is worth noticing that this operational regime does not allow to study the quantum conductance in memristors during RESET process, because of a positive feedback of device voltage during operations.[26]

Stop voltage mode in RESET process;[27,28] Chen et al.[27] and Xue et al.[28] reported another method to obtain controllable quantum conductance levels with VCM cells by varying the stopping voltage during RESET process. Nevertheless, use of STM-tip system in the fabrication process[27] makes it

difficult to be used as a component of actual devices. Importantly, the work of Xue et al.[28] reported lower control of conductance levels < $5G_0$, since the high RESET voltage can induce SET processes in the opposite polarity.

Voltage pulse stimuli operation method;[6,7,11,13,28] By appropriately tuning the voltage pulse amplitude, pulse width (width ≈ μs to ms) and pulse intervals, quantum conductance values can be obtained. The reported quantum conductance levels obtained in SET voltage pulse mode are not stable and decay rapidly after the pulse voltage is removed,[7,11] especially with small voltage amplitude to obtain low conductance, e.g., 1 G_0. Furthermore, short pulse widths and intervals (several seconds) are hard to guarantee that the filament is in an equilibrium condition, which also limits the stability of the conductance level. Due to the stochasticity of filament growth, this method does not guarantee the reproducibility of quantum conductance in both SET and RESET operational regimes.[11]

Constant voltage mode;[6,7,29,30] A constant voltage (typically some tens of mV to hundreds of mV) is applied to a resistive switch device to observe the evolution of quantum conductance levels. An electrochemical stimulus resulting from the applied constant voltage induces a conductivity change. Such conductance levels have a record stability for several hundred seconds.[29]

5. Details on the selection of materials and device configuration

On the choice of the active electrode material

Concerning the active electrode, the choice of the material relies on a balance between its chemical properties and electrochemical reactivity. First, metals with low oxygen affinity (possibly noble metals) should be preferred to avoid as much as possible chemical and/or physical interactions with the switching matrix and environment. Moreover, in this way it is possible to avoid creating oxygen chemical potential gradients, that may impact the stability in the long-term. At the same time, the metal electrode should actively respond to electrochemical stimuli. An extensive study of performance and electrochemical properties of several active metals has been reported in ref.[31] for systems based on SiO_2 dielectric layer and Pt counter electrode, in a configuration similar to our work. The properties of conductive filament formation, geometry and stability result strongly dependent on the metal Gibbs free energy (ΔG_f) for the formation of metal cations. The best conditions are found at $\Delta G_f \sim 0$, which is satisfied for Ag and Cu, the most used materials. A detailed explanation of the reasons underlying this observation and of their impact on the resistance standard application is detailed in the following.

For elements with high positive ΔG_f (such as Au), oxidation is thermodynamically unfavourable and forces the application of a high magnitude SET voltage for triggering anodic dissolution. Moreover, the low resistance state is typically observed to be unstable with significant fluctuations and spontaneous dissolution. In the context of this work, the consequent instability and reduced quantum states retention would negatively affect their programmability and reading phase.

On the opposite, for metals with negative ΔG_f (Fe, Ni, Ta, V, Ti) oxidation is significantly favoured with respect to reduction. For this reason, spontaneous interface oxidation is observed, which results in a passivation effect. The formed oxide acts as a barrier for ions injection in the switching layer, increasing the required SET voltage. The required voltage magnitude is further increased by the high energetic barrier of ions reduction reaction at the counter electrode. As a result, filaments with a wide

diameter are formed. Furthermore, the filament is expected to form a surrounding oxide layer that additionally contributes to filament stability. Consequently, high current is observed to be required for triggering the RESET process. Despite the described stabilization mechanism would be beneficial for increasing the retention of quantum states during the probing phase, it would penalize the controllability of filament programming. Indeed, the strong Joule heating caused by high RESET currents would lead to a significant increase in temperature. As described in the manuscript, temperature can be considered as the dominant control parameter determining dissolution rate and, consequently, high temperature would accelerate the filament thinning, requiring faster detection of the G_0 multiples values during the program-and-verify approach or eventually directly causing full RESET to higher conductance values. In parallel, the formation of a passivation layer would introduce an additional resistance in series to the filament one, representing an additional source of error and variability.

Metals like Ag and Cu, showing a Gibbs free energy close to zero, represent the best trade-off between oxidation and reduction affinities, forming sufficiently stable and controllable filaments under intermediate electrical stimulations. However, Cu is demonstrated to endow some detrimental properties typical of low ΔG_f materials that are not observed in Ag (which has a slightly higher Gibbs free energy). In particular, the formation of a passivation layer is observed in Cu, as confirmed through cyclic voltammetry by peak current reduction over cycling.

On the choice of the oxide layer

Regarding the oxide matrix, it also needs to fulfil specific criteria: it should be chemically stable (large deviations from stoichiometry are disadvantageous), and the metallic component should be much less mobile than oxygen to avoid competition with main mobile ions and to avoid interaction with the filament.[32] In that sense, using Al_2O_3 and Ta_2O_5 would be detrimental, as the cation transference numbers in these materials can reach up to 40 %. SiO_2 and HfO_2 appear in that sense appropriate. SiO_2 has however lower tolerance to deviation from stoichiometry. In addition, we used

8N purity SiO_2, that minimizes the probability of impurity influences, whereas HfO_2 with the same quality, for example, is not available.

6. Working principles of electrochemical polishing

Electrochemical polishing is a process developed for improving the surface quality of conductive materials. This is an approach widely used in the field of electrochemical technologies where high precision and quality of the surfaces are required.[33,34] Typically, the employed electrode process is oxidation, i.e. the material aimed to be polished is the anode. The basic principle is shown in Supplementary Figure 2.

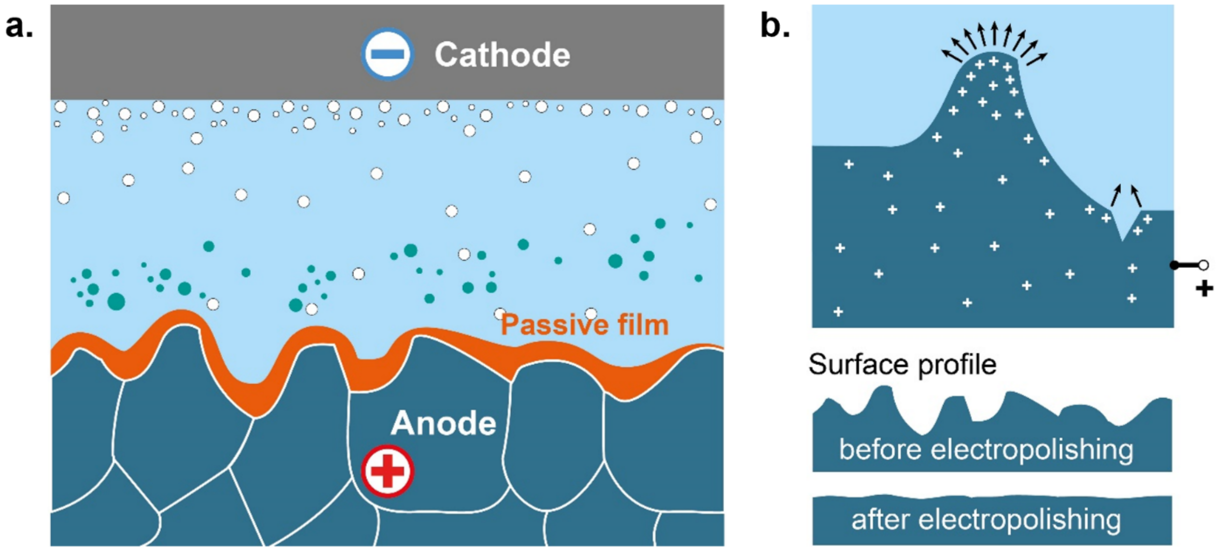

Supplementary Figure 2 | Basic principles of electrochemical polishing. a. General scheme of an electrochemical cell for electropolishing. **b.** Electric field distribution and surface profiles of the samples before and after the polishing procedure.

The initially rough anode surface is oxidized, if using materials prone to passive film formation (such as Ti, Ta, Ti, etc.). Increasing the anodic potential leads to selective dissolution of the protruded metallic peaks, due to the local increase of the electric field, and correspondingly an enhancement of the electrochemical dissolution rate in these locations. The electrochemical polishing technology results in the formation of smooth surfaces of high quality. The process, and respectively the shape and quality of the anode, can be controlled by variation of the current density, applied voltage, electrolyte composition/concentration and temperature. In case that materials should be polished not forming oxides in the selected voltage region (i.e., noble metals) one can use additives that adsorb at

the electrode surface to form a passive film, similarly to the effect of the oxidation of non-noble metals.

7. Electrochemical polishing effect in memristive devices

While the direct visualization of electrochemical polishing effects in nanoscale filaments is extremely challenging since current state-of-the-art technology is not allowing direct imaging of nanoscale objects with atomic resolution (this would also pose several challenges in the sample preparation), as required to observe the dissolution of single atoms of atomic chains, signatures of the electrochemical polishing effects can be observed in the electrical response of the device. This effect can indeed explain experimental observations of *i)* gradual RESET with discrete levels versus abrupt SET, *ii)* decrease of the low conductance state by extending the negative voltage sweep range, *iii)* reduction of the low conductance state variability over cycling, *iv)* filament polishing in a small voltage window, as detailed in the following.

i) Gradual RESET versus abrupt SET

When comparing the current-voltage behaviour during SET and RESET, it becomes obvious that the formation of the filament (SET) is an abrupt process that cannot be controlled. The growth of the filament occurs under conditions of high electric fields and current densities. As the filament is growing, the electric field even increases, due to the decreasing distance between the filament and electrode (at the same applied voltage). Despite the main charges determining the measured currents are electrons, the ionic current cannot be neglected. Electrochemical reactions and ion transport are field accelerated and exponentially dependent on the field. These conditions lead to a situation where the growth of the filament cannot be effectively controlled and tailored.

In contrast, the process of dissolution of the filament is not abrupt but nearly gradual. Despite the initial total currents being comparable to the SET currents, they are practically almost purely electronic. This leads to a pronounced heating (Joule effects), however no exponential acceleration of the redox reactions and ionic transport is present. According to the principle of electrochemical polishing, the electrochemical oxidation/dissolution starts with the atoms/clusters from the filament that are less stable and with high surface free energy (the process is supported by the locally increased

temperature, lowering the required oxidation potential) and then continues to the more stable core of the filament, until it finally breaks the contact.

The above-discussed effects can be observed in the *I-V* characteristics reported in Supplementary Figure 3 where, in contrast with an abrupt SET, a gradual RESET process can be explained through the electrochemical polishing effect that progressively removes less stable atoms, progressively shrinking down the filament size. Analysing this behaviour, it can be concluded that filament strength and size can be much better controlled during the RESET (dissolution), where it is possible to exploit the electropolishing effect. Limiting the RESET voltage to less negative values leads only to the removal of unstable atoms and needles from the filament, but not dissolving its core (partial RESET).

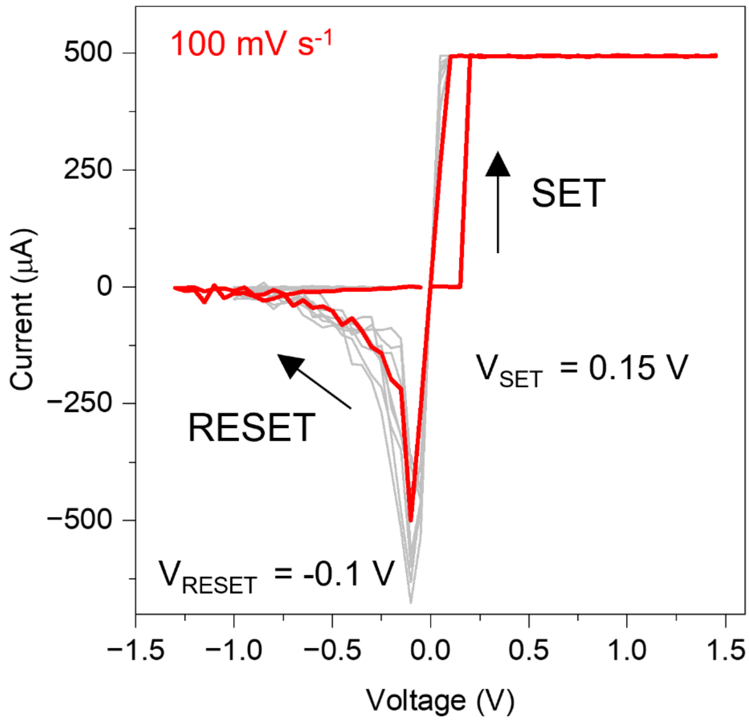

Supplementary Figure 3 | Typical resistive switching characteristics of Ag/SiO_2/Pt memristive devices. The hysteretic *I-V* resistive switching curve under voltage sweep stimulation is characterized by an abrupt current transition during SET process (here, a current compliance of 500 μA is applied to prevent hard breakdown of the device) and by a gradual current transition during RESET process exemplifying the electrochemical polishing effect. The *I-V* characteristics were obtained by driving the device with a voltage sweep rate of ~100 mV/s.

ii) Modulating the high resistance state by extending the negative voltage sweep range

Supplementary Figures 4a and b report switching cycles obtained by progressively extending the range of the negative voltage sweep used to RESET the device. For each negative voltage range, 5 cycles after stabilization are reported, since transient dynamics characterized by unstable behaviour can be observed after changing programming conditions (details in the following section iii). According to the principle of electropolishing, atoms and atomic chains connected to the main filament body and characterized by higher surface energy will be firstly removed when higher energy is provided (e.g. thermal or electrical). This process is progressively enhanced when extending the negative voltage range, leading in turn to a progressive reduction of the filament size. As a consequence, this leads to a progressive lowering of the device conductance. According to the principle of electropolishing that would imply the removal of a progressively higher number of unstable atoms when higher energy is provided, a progressive reduction of the filament size by extending the negative voltage range results in a decrease of the low conductance state of the device. Notably, the reduction of the filament size drives the device into the quantum conductance regime (conductance of few G_0), revealing that the effect of increasing the negative sweep range is the discrete, progressive reduction of the filament size by few (less stable) atoms. Also, it is important to say that in the considered negative voltage sweep ranges, the device did not experience a hard RESET with complete rupture of the filament, since the RESET process was characterized only by a narrowing of the filament without complete rupture (partial RESET).

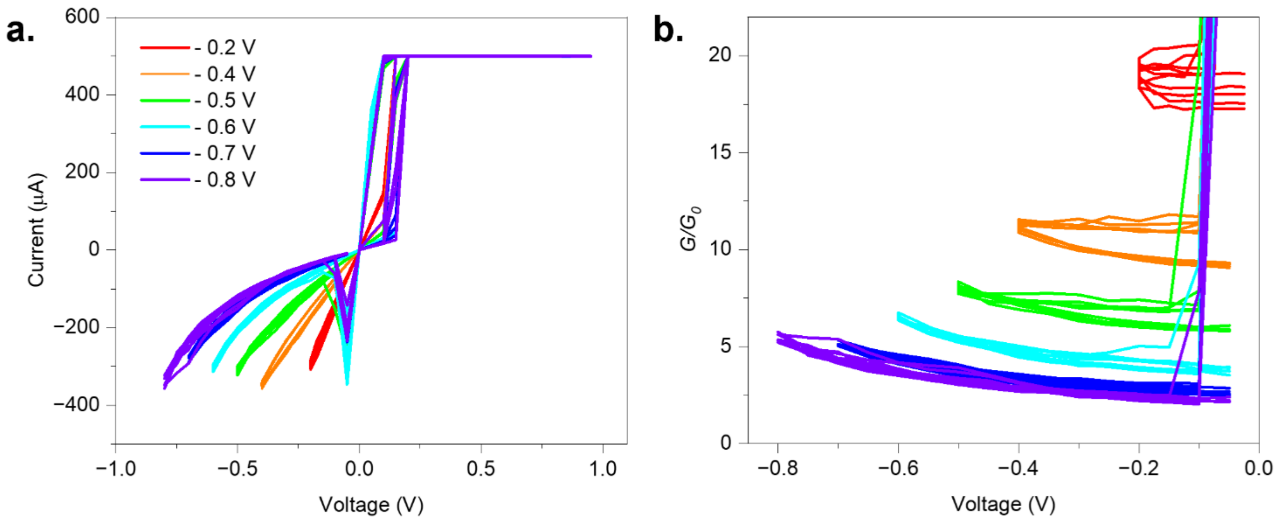

Supplementary Figure 4 | Decrease of the low conductance state by extending the negative voltage sweep range. a. *I-V* characteristics of the device obtained by progressively extending the negative voltage sweep range and **b.** corresponding conductance over cycling. Results have been obtained by progressively extending the negative voltage sweep range from -0.2 to -0.8 V, keeping fixed all other conditions (CC = 500 µA, sweep rate of 300 mV/s). For each negative voltage range, 5 cycles after device stabilization are shown. Indeed, transient dynamics leading to a more unstable behaviour are observed immediately after the increase of the negative voltage sweep until the filament organizes itself in a new stable configuration.

iii) Adjustment of the low conductance state over cycling

Supplementary Figures 5a and b report the evolution of the *I-V* resistive switching characteristic over cycling, showing that the low conductance state is stabilized over cycling. This can be interpreted in terms of the electrochemical polishing effect that strips out the excess of atomic chains over cycling, leading to a more stable nanofilament configuration. First cycles remove most unstable atoms, but this process is not occurring in one step, but needs several tens of cycles, until a stable steady state (resistance) is reached.

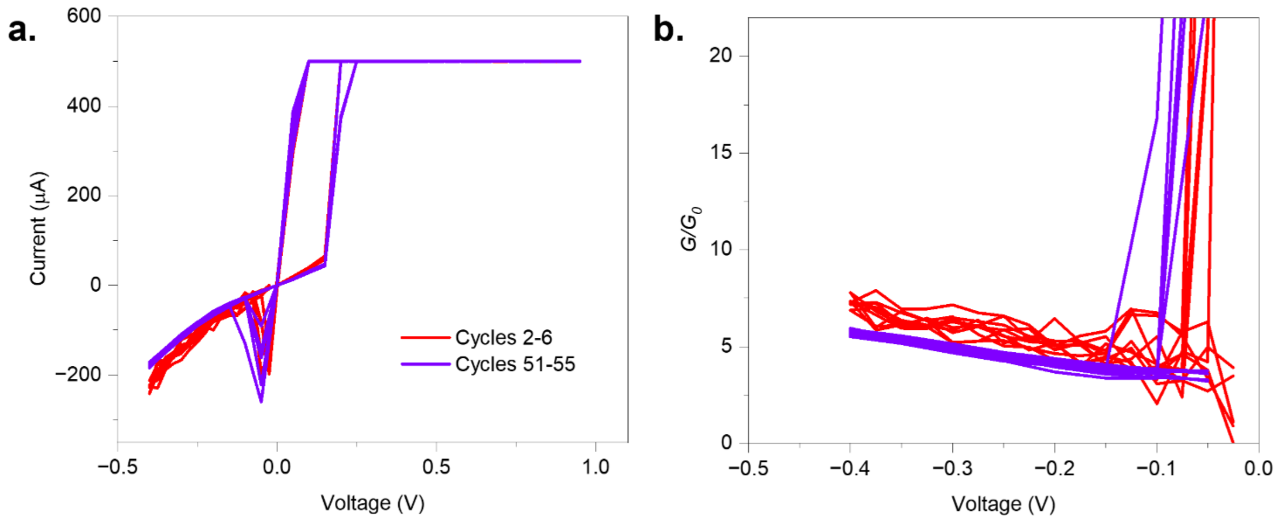

Supplementary Figure 5 | Reduction of the low conductance state variability over cycling. a. *I-V* characteristics and **b.** corresponding conductance of the device during cycles 2-6 and during cycles 51-55, showing that the low conductance state tends to stabilize over cycling.

iv) Filament polishing in a small voltage window

Another demonstration of electrochemical polishing effect acting on the nanofilament can be observed when applying narrow voltage window sweeps and record the electrical response of the device. After cycling the device using a negative voltage sweep range that leads a partial RESET (i.e. the reduction of the metallic filament without complete rupture) (Supplementary Figure 6a), we have reduced the cycling voltage window to 300 mV. Supplementary Figure 6b-e report the evolution of the electrical response of the device when continuously stimulated with voltage sweeps in a small voltage range, i.e. in a voltage range that do not allow to SET and RESET the device and at the same time is asymmetric in respect to the zero (200 mV in negative and 100 mV in positive direction). As can be observed, the electrical response in cycles 1-10 (Supplementary Figure 6b) is showing higher variability in conductance values, whereas in cycles 11-20 (Supplementary Figure 6c) the steady state value is almost reached. Consequently, the distribution of conductance in cycles 1-10 (Supplementary Figure 6d) is larger than in cycles 11-20 (Supplementary Figure 6e). This is consistent with the electrochemical polishing interpretation where the small voltage sweeps result in a progressive

polishing of the nanofilament to strip out less stable atoms, progressively leading to a more stable nanofilament configuration.

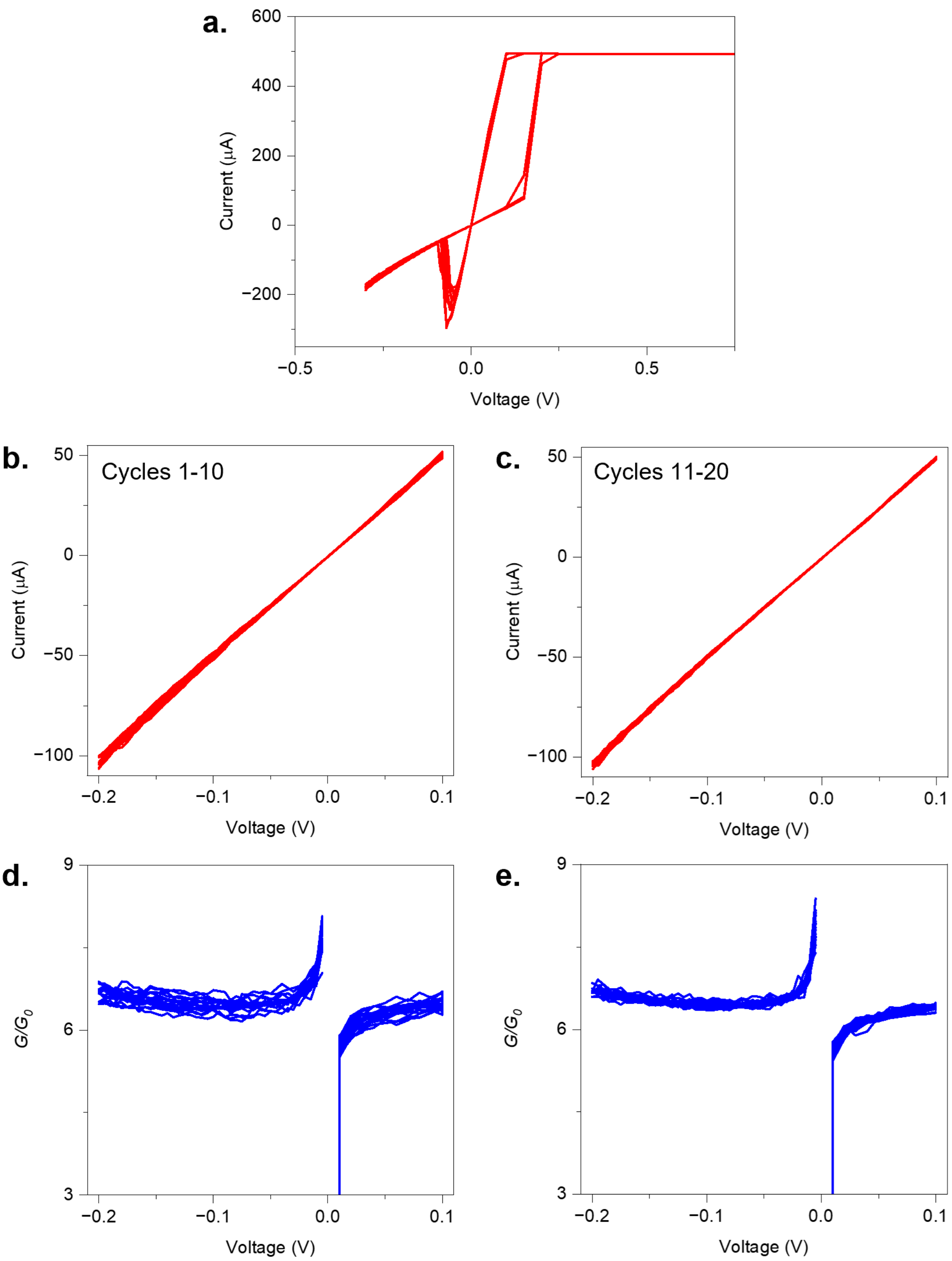

Supplementary Figure 6 | Small voltage window electrochemical polishing. a. Resistive switching characteristics of a device allowing to obtain a partial RESET (negative voltage range of -0.3 V), leading to the reduction of the metallic filament size without a complete rupture. After cycling, the device was left in the low conductance state and then stimulated with a voltage sweep in a smaller voltage window (+0.1 V, -0.2 V), not allowing the device to SET or RESET. *I-V* characteristic of the device when stimulated with a voltage sweep in a small voltage window during cycles 1-10 and cycles 11-20 are reported in panels **b** and **c**, respectively. Corresponding conductance traces are reported in panels **d** and **e**, respectively. As can be observed, a progressive reduction of the conductance distribution can be observed over cycling the device in a small voltage window.

8. On the electrochemical polishing effect in ECM and VCM cells

The electrochemical polishing effect can be applied to memristive devices relying on the electrochemical metallization mechanism (ECM), as in our case, where filaments are metallic and polishing leads to selective dissolution of unstable atoms and/or atomic chains at the surface of the filament. Instead, in case of devices based on the valence change mechanism (VCM) where the filaments (in the general case) are composed of reduced oxide, the anodic potential will not necessarily lead to removal of atoms but rather to further oxidation of the filament and/or to changes in the interfacial Schottky barrier.

In the case of VCM-based memristive devices, the mobile species are oxygen ions, where the redox reaction is changing the oxidation state of atoms and cations, but these mobile species are not moving themselves. Therefore, strictly speaking, it is not possible to say that electrochemical polishing can occur in this case. The stoichiometry (composition) of the filament is changed and not its morphology. In such systems, composed from reduced oxide (or metal such as Hf or Zr with high oxygen affinity), there is a strong chemical potential gradient of oxygen, aiming to completely oxidize the reduced part. The high stability of the filaments (and conductance states) can be attributed to kinetically frozen/retarded processes, i.e. the driving force for complete oxidation is present, but the kinetics is so slow at the concrete conditions, that transport of ions (without applied voltage) and reaction rate are extremely slow. For example, these principles are at the base of the controllability and stability of quantum conductance levels in Pt/HfO_x/ITO memristors.[28] While in HfO_2 dominating mobile species are oxygen ions (Hf-ion mobility can be almost neglected), in other oxide systems such as for example Ta_2O_5, or Al_2O_3 both cations and anions can be mobile.[32] In this case applying a voltage will lead to movement of both the cations and anions in different directions, leading to less controllable states.

In the case of Pt/SiO_2/Ag ECM system (our study), we remove Ag atoms directly from the filament. Thus, the stoichiometry of the filament is not changed, but only the atoms/atomic chains that have

higher excess surface energy (and contribute to the total conductivity) are removed. Here we can claim for the electrochemical polishing effect. In addition, Ag is a noble metal, and it does not form easily stable oxides, and the system is thermodynamically stable.

9. Details on the modeling approach

Although the presented model is inspired on the physics of the resistive switching mechanisms (i.e. electropolishing during the RESET transition), this is a compact behavioural model based on simple assumptions for the considered equations and dynamic parameters. The goal of such a model is to reproduce the electrical behaviour of the device under any kind of input signal. In our case, the model is based on two equations (Eq. 1 and Eq. 6 in Methods). Eq. 1 is for the state variable (chosen to be the number of conducting channels, n_{ch}) which is related to ionic motion and chemical reactions. Eq. 6 is for the electron transport, which is related to n_{ch} as well. The two basic equations of the model contain several parameters which are used to quantitatively reproduce the electrical response of the devices. Fitting of experimental results allows to extract these parameters. In this work, we have used I-V loops since these are the data available from the inter-laboratory experiment. A complete set of pulsed measurements with different voltage amplitudes would allow, for example, an equivalent extraction procedure. Having determined the values of the parameters, we have shown that the model nicely captures the main trends of the experimental results, as shown in Figure 2c and d.

For a more concrete discussion, let us focus on the RESET transition, which is the one of interest in this work. This transition consists in the voltage-induced change of the filament conductance from $G_{init} = n_{init}G_0$ (the value reached at the end of the previous SET transition) to the $2G_0$ and G_0 states. The RESET transitions are described by Eq. 5 and Eq. 6 in Methods. Let us examine these equations in detail and discuss about the involved parameters and the underlying physics.

In Eq. 5, n_{max} is the maximum number of conducting channels. As stated above, n_{max} is proportional to the maximum area of the filament which, unfortunately, cannot be directly measured. Thus, n_{max} is a heuristic model parameter. On the other hand, $\tau_R(V)$ is the characteristic RESET time which must certainly depend on voltage, current and temperature. In our model, this characteristic time has been considered to depend on four parameters. Namely, the scale parameter τ_{R0}, the activation energy E_{max} and the two components of the thermal resistance, R_L and R_T.

Moreover, the effectively applied voltage depends on the series resistance R_S. In total, the model has 6 parameters, and it provides a reasonable framework to describe the electrical properties.

Let us examine the foundations and limitations of each of these parameters. First, we have considered that $\tau_R(V)$ depends on the applied voltage (and on the current) because it determines the dissipated power and hence, the temperature. However, in the general case, other mechanisms such as the drift of metal atoms towards the cathode might also be relevant. Consideration of an expression of the type $\tau_R(V) \sim \exp(-\gamma V) \exp[-E_{act}/K_B T_{CF}(V,T)]$, with γ being a voltage acceleration parameter similar to that considered for the SET transition, might improve the description of the actual physics. Moreover, this could provide a more flexible model to fit the experimental results. However, this would include more parameters and increase the model arbitrariness. As for the temperature dependence, and Arrhenius dependence on the local filament temperature has been assumed. This is a first-order high-level representation of relevant phenomena such as the diffusion of metal atoms and the redox reactions involved in the electropolishing mechanism. However, the detailed description of these phenomena is far from being easy and hence, complicating the description is unnecessary. Moreover, the effects of power dissipation are included through a phenomenological thermal resistance (with two parameters) in agreement with previous works[35,36]. This is certainly an oversimplification, and a detailed solution of the heat equation would be required for a sounder description of the involved physics. Finally, the existence of a series resistance, R_S, is undisputable. This resistance is expected to have two components, an external resistance related to the measurement setup and the metal contact resistances, and an internal resistance related to the access region (in the conductive filament itself) to the constriction that determines the transport. Both components are unavoidable and cannot be directly measured. Thus, again, this is an unknown parameter. On the other hand, the transport equation, Eq. 6, can be simplified by eliminating the second term, as discussed above. When there is a conducting channel, the underlying tunnelling conduction is

negligible, and the current is proportional to the number of conduction channels, n_{ch}. Thus, Eq. 6 does not introduce any extra parameter.

In conclusion, our approach is that of a behavioural compact model that is intended for circuit simulation (i.e. SPICE). The model is valid for any type of input voltage waveform (voltage ramps, current ramps, constant voltage, constant current, pulsed voltages…) without any modification of the involved parameters.

By considering selected I-V loops obtained by different partners during the interlaboratory comparison (with the same measurement protocol characterized by stimulation of the device with a voltage sweep rate of 96 mV/s and 2 mV/s during the SET and RESET sub cycles, respectively, and current compliance of 500 μA during SET operation), a strong correlation between the conductance reached at the end of the SET transition and the shape of the RESET transition is observed. This correlation can be represented by the dependence of the RESET current peak on the conductance at the beginning of the RESET cycle. Supplementary Figure 7 demonstrates that the model can capture this experimental correlation by changing the value of n_{init}. This gives strong support to our modelling approach and reveals that the characteristic RESET time is accelerated by the local conductive filament temperature (power dissipation) rather than by the electric field.

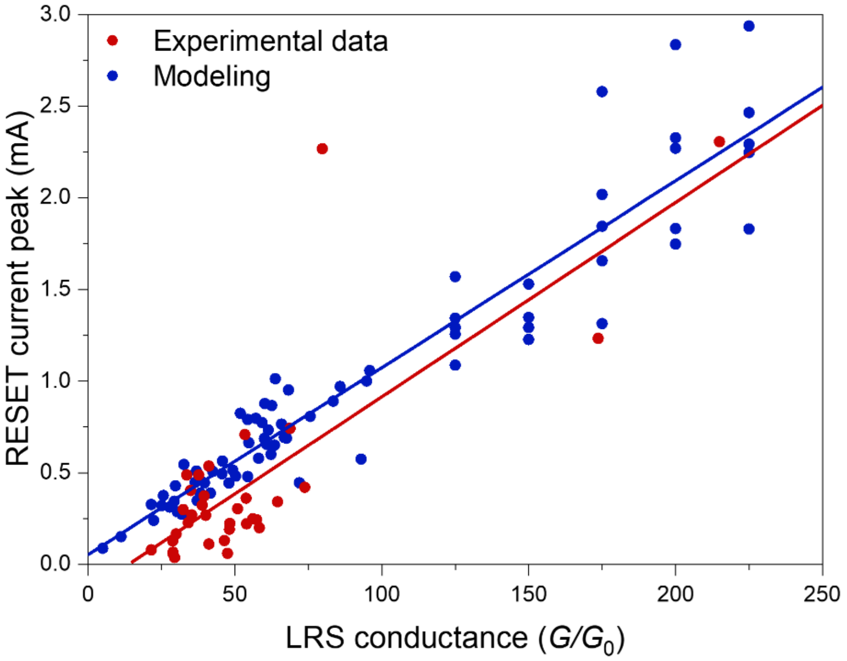

Supplementary Figure 7 | Correlation between current peak during RESET and low resistance state (LRS) of the device after SET. Comparison of experimental results and modeling.

10. Experimental and modeling RESET characteristics

Supplementary Figure 8a shows examples of experimental *I-V* RESET characteristics obtained during the interlaboratory comparison (data from different labs), while Supplementary Figure 8b shows results from the stochastic modeling. As can be observed, the model can reproduce the variability of experimental results.

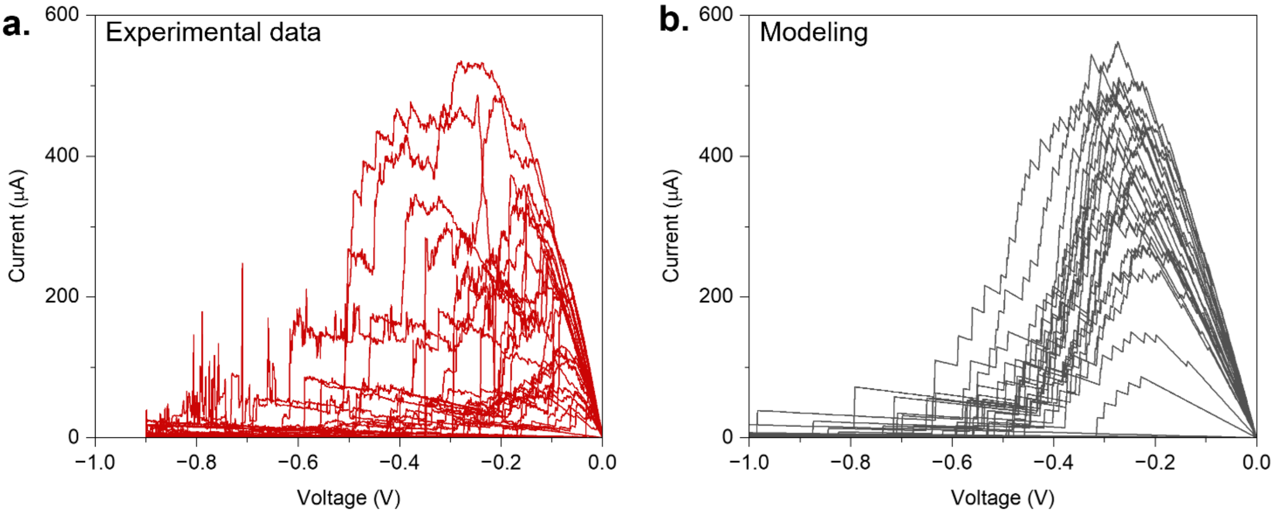

Supplementary Figure 8 | Experimental and modeling RESET characteristics. a. Selected experimental curves obtained during the interlaboratory comparison (curves from different labs) and **b.** Results obtained by modeling, here a Gaussian distribution of n_{init} was employed to cover the full range of situations at the beginning of the RESET cycle. In both panels, current is represented in absolute value.

To complement the results shown in Figures 2c and 2d, we report in Supplementary Figure 9 examples of experimental and modeling curves for the *I-V* RESET transition and the related conductance transients at the final stage of the RESET. Of course, being a stochastic approach, the exact values of conductance and the time location of the conductance jumps cannot be matched to experiments. However, these results show that the model can capture the main trends of the experimental RESET transitions.

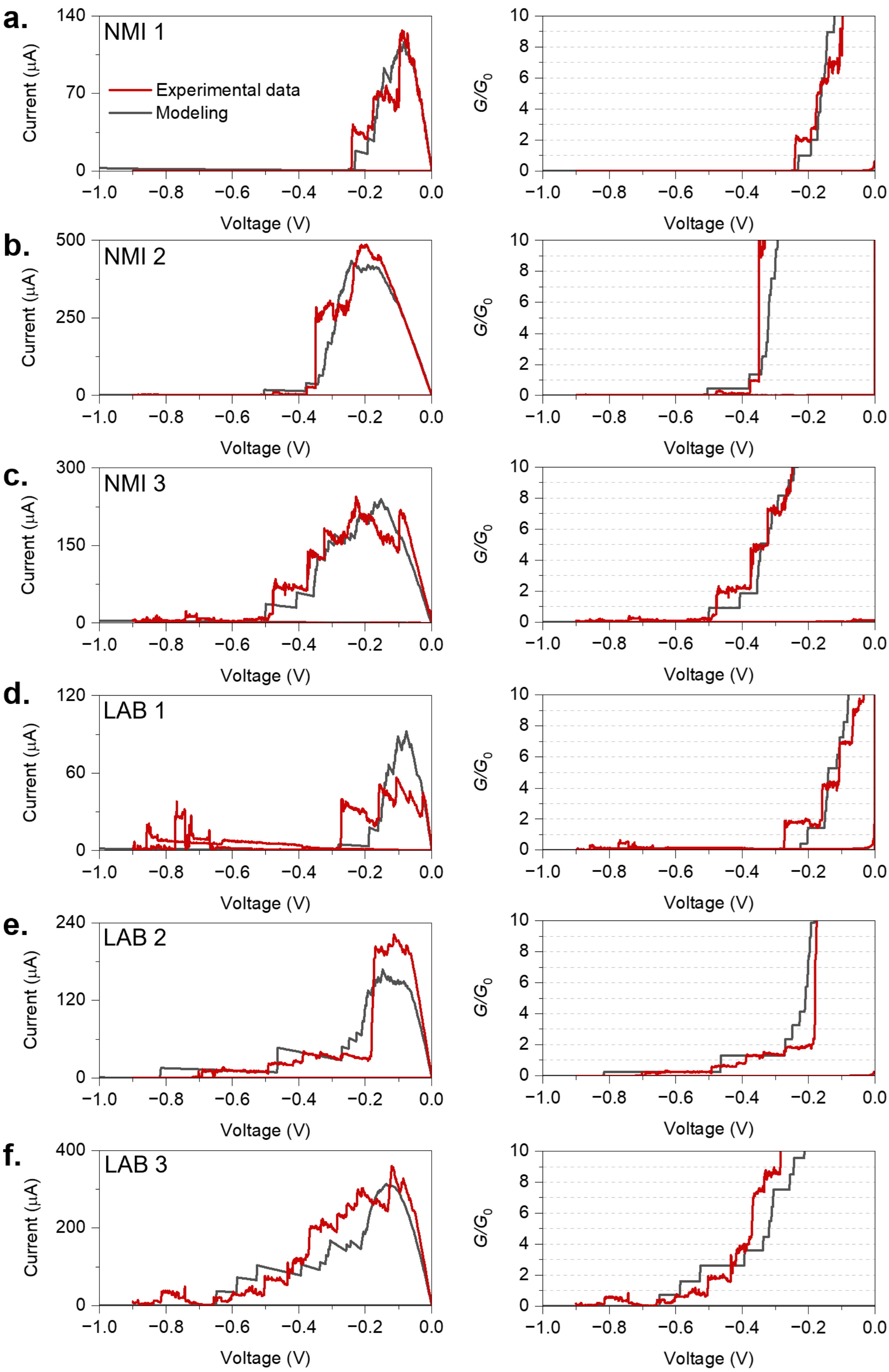

Supplementary Figure 9 | Fitting RESET transitions from different laboratories. a-f. Fitting of selected experimental *I-V* characteristics obtained by different labs with the same measurement protocol. Red lines are experimental results, black lines are model simulations. Left panels show the RESET *I-V* characteristics, right panels the evolution of conductance at the end of the RESET process.

11. "Program and verify" approach for practical realization of a voltage standard based on Josephson effect

DC Voltage standards based on the Josephson effect have been used as voltage primary standard for decades.[37] Its operation relies in the activation of junctions of superconductor-insulator-superconductor materials in an array. The generated DC voltage across the array corresponds to quantum values multiples of the Josephson constant (K_J = 2e/h), $V_n = n f / K_J$, where n is an integer related to the number of activated junctions and f is the microwave frequency applied to the device. The process to activate the junctions is achieved through the application of a sweep voltage between zero and the desired voltage step value V_n for several cycles. The amplitude of the sweep voltage will control the number n of activated junctions and therefore the desired quantum voltage step. When this sweep signal is stopped, a number n of junctions will remain activated, and the corresponding voltage is fixed at the device terminals. To confirm the success of the process and the right number of activated junctions, the voltage at the device terminals is roughly measured to identify the corresponding nearest integer n which easily reveals the exact Josephson Voltage step. For example, if f = 70.0 GHz, $V_{n+1} - V_n \approx 145$ µV and this means that a measure of V_n with a resolution of half of 145 µV is enough to allow the identification of the achieved step. If that does not correspond to the desired step, the process of activation is repeated and checked again.

12. On the stability of quantum conductance values

Supplementary Table 1 reports a comparison of obtained results with literature on memristive devices operating in the quantum conductance regime in terms of retention of the G_0 state, comparing also device structure, switching mechanism and programming approach.

Supplementary Table 1. Comparison of retention times of the quantum conductance state related to G_0 in memristive devices reported in literature. The table includes also work reporting the observation of quantum conductance levels not showing retention of the quantum conductance level corresponding to 1 G_0. This includes studies in which the fundamental quantum is not observed (n.o.), or its temporal behavior is not reported/cannot be retrieved from reported data (n.r.).

Reference	Device Structure	Switching Mechanism	Programming approach	*G₀* retention [s]
Wagenaar et al. [38]	Ag/Ag2S/Pt	ECM	Constant voltage (reset)	n.o.
Zhu et al. [39]	Nb/ZnO/ITO	ECM	Voltage sweep (set)	n.r.
Zhu et al. [39]	ITO/ZnO/ITO	VCM	Voltage sweep (set)	n.r.
Mehonic et al. [40]	Poly-Si/SiOx/p-type Si	VCM	Voltage sweep (reset)	n.r.
Nandakumar et al. [41]	Cu/SiO2/W	ECM	Current sweep (set)	n.r.
Maudet et al. [42]	Cu/SiO2/W	ECM	Voltage sweep (set)	n.r.
Tappertzhofen et al. [43]	Ag/AgI/Pt	ECM	Current sweep (set)	n.r.
Yi et al. [44]	Ta/TaOx/Pt	VCM	Current sweep (set and reset)	n.r.
Long et al. [45]	Pt/HfO2/Pt	VCM	Voltage sweep (set)	n.r.
Krishnan *et al.* [46]	Ag/PEO/Pt	ECM	Voltage sweep (set)	≤ 2
Chen *et al.* [47]	Ti/Ta_2O_5/Pt	VCM	Pulsed voltage (set and reset)	2*
Younis *et al.* [48]	Au/SnO_2–CeO_2/F-SnO_2	VCM	Pulsed voltage (set and reset)	3*
Yi et al. [44]	Ta/TaOx/Pt	VCM	Pulsed voltage (set)	60
Tsuruoka *et al.* [49]	Ag/Ta_2O_5/Pt	ECM	Pulsed voltage (set)	60
Zhao *et al.* [50]	Mg/Pectine/Mg	ECM	Pulsed current (set)	100
Kharlanov *et al.* [51]	Cu/PPX/ITO	ECM	Pulsed voltage “Write-verify”	300*
Deswal *et al.* [52]	Al/Nb_2O_5/Pt	ECM	Voltage sweep (set)	500
Köymen *et al.* [53]	Au/Cr/TiO_x/TiO_2/Cr/Au	VCM	Voltage sweep (reset) “Program and verify”	4000
Xue *et al.* [54]	Pt/HfOx/ITO	VCM	Pulsed voltage (reset)	7000
Banerjee *et al.* [55]	Cu/Ti/HfO_2/TiN	ECM	Pulsed voltage (set)	1e4
Chen *et al.* [56]	Pt/HfOx/Pt	VCM	Voltage sweep (reset)	1e4
**This work**	**Ag/SiO_2/Pt**	**ECM**	**Voltage sweep (reset) “Program and verify”**	**1.6e4**

* Retention values limited by intentional conductance state reprogramming.

13. Evaluation of repeatability of the quantum conductance value

The conductance value for each quantum level has been evaluated using an average of multiple consecutive measurements, after an initial stabilization period of 10 measurements (an example is reported in Supplementary Figure 10). For this purpose, additional to the condition of the measured values remain in the interval in the intervals [0.5 G_0; 1.5 G_0] or [1.5 G_0; 2.5 G_0], a minimum of 30 to a maximum of 100 consecutive measurements were considered as fixed conditions in the data evaluation. Depending on the experimental setup and its measurement parameters (as NPLC – Number of Power Line Cycle, Filter, etc.), the acquisition period of consecutive data is in the range ~ 10 – 120 s. The variability over these consecutive measurements has been exploited to evaluate repeatability (by the corresponding standard deviation) of the quantum conductance level.

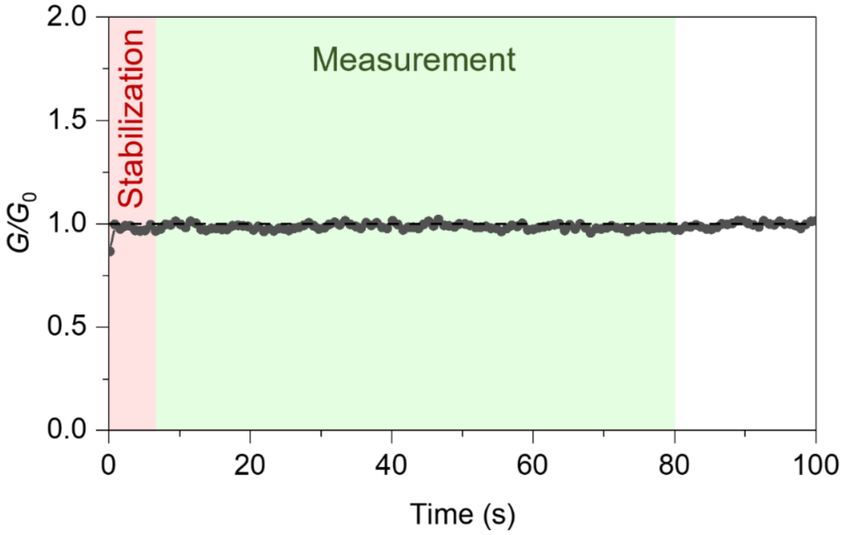

Supplementary Figure 10 | Evaluation of repeatability of the quantum conductance value. The repeatability has been evaluated based on consecutive measurements (100 measurements in this case) while applying a constant reading voltage of the device of 10 mV, discarding the initial 10 measurements where device stabilization can occur.

14. Specification of equipment and corresponding measurement accuracy

Supplementary Table 2 reports instrument specifications in the measurement conditions exploited for the measurement of quantum conductance level values, and the corresponding measurement accuracy exploited for the evaluation of the measurement uncertainty related to the measurement equipment.

Supplementary Table 2 | Specification of equipment and corresponding measurement accuracy.

Participant	Equipment	Voltage accuracy ± (%reading + volts)	Current accuracy ± (%reading + amps)
**Specification corresponding to 1 μA range used in the G_1 group of measurements**			
LAB 1	Keithley 6430	0.012 % + 350 μV	0.05 % + 300 pA
LAB2	Keithley 4200 SCS-AC	0.012 % + 100 μV	0.05 % + 100 pA
NMI 1			
LAB 3			
NMI 2	Keithley 2400	0.012 % + 300 μV	0.029 % + 300 pA
NMI 3	Keysight B1500A	0.01 % +120 μV	0.05 % + 100 pA + 0.1%V_0
**Specification corresponding to the 10 μA range used in the G_2 group of measurements**			
LAB 1	Keithley 6430	0.012 % + 350 μV	0.05 % + 2 nA
LAB2	Keithley 4200 SCS-AC	0.012 % + 100 μV	0.05 % + 600 pA
NMI 1			
LAB 3			
NMI 2	Keithley 2400	0.012 % + 300 μV	0.027 % + 700 pA
NMI 3	Keysight B1500A	0.01 % +120 μV	0.04 % + 2 nA + 1%V_0

15. Statistical validation of the programming methodology

A key claim of this work is that there are conductance states related to the quantum of conductance G_0 and that these states can be programmed to implement a standard of resistance only related to universal constants of nature. To this purpose, a program and verify procedure which selects only a conductance state G in the $0.5G_0 \leq G \leq 1.5G_0$ range (in case of G_1) was proposed. However, this procedure needs to be statistically validated. In the present discussion, we focus on the analysis of the quantum conductance level related to G_0 state, but we have checked that the case of the quantum conductance plateaus related to $2G_0$ is fully equivalent. Our aim is to analyze the measured statistical data (117 conductance values related to G_1 obtained in different laboratories, refer to Extended Data Figure 3) to confirm that there is a preferred conductance state around G_0.

The main objection that can be made to the methodology of programming the conductance states with the program and verify procedure is that it will always select conductance states in the established range, no matter whether there is a real conductance peak around G_0 or not. The question is that even in the extreme case of a uniform distribution of conductance in the considered range, the mean of the statistical distribution would be G_0.

Let us assume, for the time being, that there are preferred atomic-size configurations of the filament with a conductance $\sim G_0$. Of course, a dispersion around this value is expected because slightly different structural dispositions of the filament atoms would give rise to different transmission coefficients. We will make the analysis in terms of the normalized conductance $x = G/G_0$ and we start with the hypothesis that there is an intrinsic gaussian distribution (the parent distribution) with mean value $\overline{x_p} = 1$ and standard deviation σ_p. The terms "intrinsic" or "parent" mean that no external selection constraints (no "program and verify" process) are applied.

Assuming that there is an intrinsic gaussian peak and applying a program and verify procedure that establishes lower and upper conductance boundaries $(1 - \Delta x < 1 < 1 + \Delta x)$, we expect to find a

truncated gaussian distribution in the experimental results, Δx being the truncation or censoring interval. If $\sigma_p \ll \Delta x$, the experimental distribution would be equal to the parent distribution, but otherwise, the truncated distribution would not be Gaussian and, in the limit $\sigma_P \gg \Delta x$, it would converge to the uniform distribution. This is illustrated in Supplementary Figure 11 where the parent probability density function is compared with that of the truncated distribution for three different values of σ_p which almost cover the full range from the gaussian to the uniform distributions.

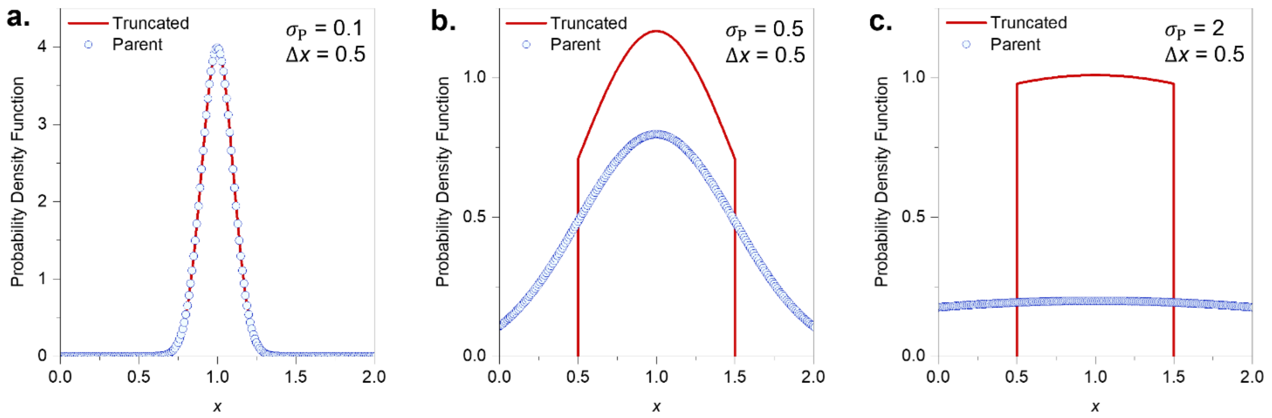

Supplementary Figure 11 | Comparison of parent and truncated distributions. **a-c.** Comparison of parent and truncated distributions for different values of the standard deviations and the same truncation interval $\bar{x} \pm 0.5$.

In the experiments, we have considered $\Delta x = 0.5$, and the mean and standard deviation directly obtained from the data are $\bar{x}_{exp} = 0.96$ and $\sigma_{exp} = 0.23$. The analysis in terms of the Gaussian distribution, $\Phi(x)$ can be performed with the normalized $\Phi^{-1}(F)$ versus x plot, and the extracted parameters are $\overline{x_G} = 1$ and $\sigma_G = 0.24$. On the other hand if the distribution were uniform, the expected parameters would be $\overline{x_u} = 1$ and $\sigma_u = 2\Delta x/\sqrt{12}$ so that, for $\Delta x = 0.5$, $\sigma_u = 1/\sqrt{12} \sim 0.29$. While the parameters extracted from the gaussian analysis coincide with the experimental results, there is a discrepancy between σ_u and σ_{exp}. This discrepancy is consistently observed in the data of the different laboratories and for the conductance state related to $2G_0$, as shown in Supplementary Figure 12a.

On the other hand, we have randomly simulated the truncated distribution by emulating the program and verify procedure as a function of σ_p. In Supplementary Figure 12b, we show the standard deviation of the parent distribution as a function of σ_p and we compare the results with those expected for purely gaussian and purely uniform distributions. According to these results, the experimental value, $\sigma_{exp} = 0.23$, would correspond to a gaussian distribution with $\sigma_p = 0.3$. Notice that, consistently to what is qualitatively shown in Supplementary Figure 11, the truncated distribution converges to the uniform/gaussian distributions for wide/narrow parent distributions, respectively. Moreover, it is also evident that our experiments are in the transition region between these two limits.

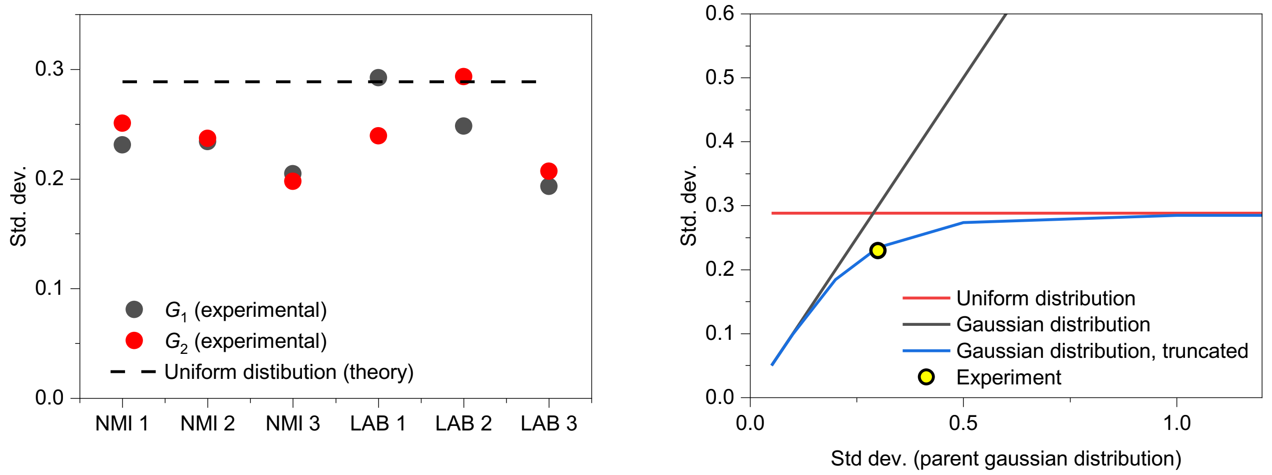

Supplementary Figure 12 | Comparison of experimental results with gaussian and uniform distributions. a. Comparison of the values of the standard deviation σ_{exp} obtained in all the laboratories involved in the interlaboratory experiment with the value expected for a uniform distribution. **b.** Standard deviation of the truncated distribution as a function of the standard deviation of the assumed gaussian parent distribution. The limits of purely gaussian (narrow parent distribution) and purely uniform (wide parent distribution) are also shown for comparison. The experimental point is found in the transition between the two limits.

Having concluded that the experiments are consistent with a gaussian parent distribution with $\sigma_p \sim 0.3$, we can now study the impact of the censoring interval Δx through simulations of the truncated distribution. This is a complementary way to look at the same subject represented in Supplementary Figure 12b. Now, σ_p is kept fixed at 0.3 and Δx is the independent variable. As shown in Supplementary Figure 13a, the truncated distribution converges to a purely gaussian at large values of Δx and to a uniform distribution for small values of Δx. Again, the experimental results are in the

transition region between both limits. As previously mentioned, although the difference between σ_{exp} and σ_u is not very large, it is consistent with our analysis of the truncated distribution and can be assumed to be significant to confirm that the probability density distribution has a peak in the considered conductance range.

While we cannot change the standard deviation of the parent distribution because it is an intrinsic property of the conductance statistics, we can decide which the truncation interval is. However, there are limits related to programming efficiency and overlapping between conductance peaks. If Δx is increased, the overlapping with the following peak (in our case with the $2G_0$ peak) increases. We have estimated that the overlapping with the $2G_0$ peak is about 5% for $\Delta x = 0.5$, a value that is very reasonable given the rather large value of the standard deviation of the parent distribution as compared with the separation between the conductance peaks. Moreover, increasing the censoring interval would increase the standard deviation of the experimental results which is undesirable for the application of a resistance standard. On the other hand, if Δx is decreased, the standard deviation of the experimental distribution would decrease (something that is good for the resistance standard) but the number of RESET cycles required to reach the desired level would increase. It can be demonstrated that this number of cycles scales with $1/(\Phi(1+\Delta x) - \Phi(1-\Delta x))$ and this explodes below $\Delta x = 0.25$, as also shown in Supplementary Figure 13a. Thus, there is still room to decrease the standard deviation of the resistance standard by choosing $\Delta x < 0.5$. However, as shown in Supplementary Figure 13a, the discrimination between the gaussian and the uniform distributions would be even more difficult for smaller values of Δx. Thus, $\Delta x = 0.5$ is a rather optimal choice to obtain a reasonable standard deviation of the experimental results, to keep the programming efficiency under control and to discriminate between gaussian and uniform distribution, providing evidence of the existence of the intrinsic G_0 (and $2G_0$) conductance peaks.

Finally, Supplementary Figure 13b shows the parent and truncated distributions for the case of $\sigma_P =$ 0.3. Notice that, in this case, the truncated distribution is close to gaussian. This is the reason why the analysis of the experiments in terms of a gaussian distribution provides very good results.

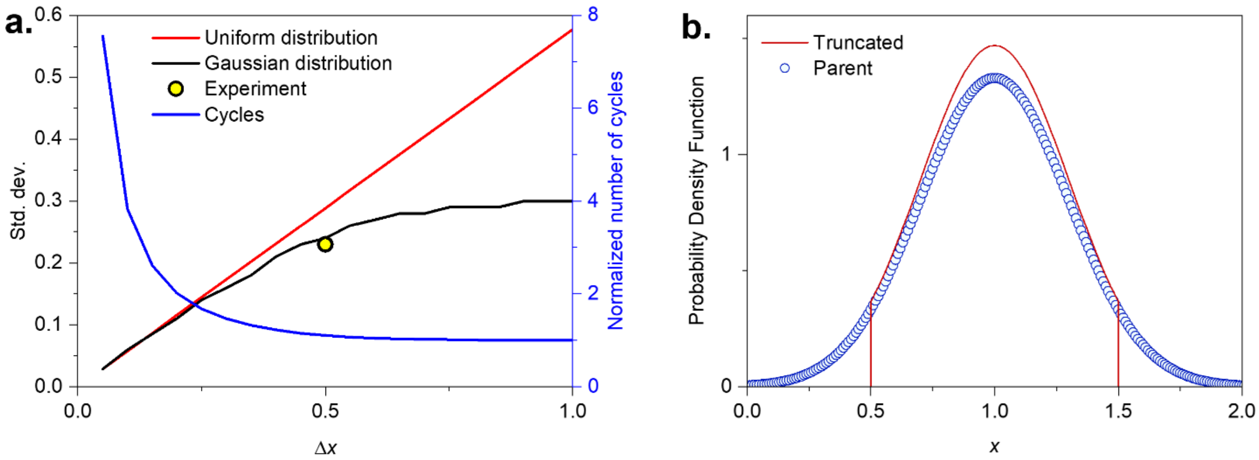

Supplementary Figure 13 | Effect of the censoring interval in the program and verify approach. **a.** Standard deviation of the truncated distribution as a function of the censoring interval Δx: gaussian parent distribution with $\sigma_p = 0.3$ (black); uniform distribution (red); experimental result (yellow dot) and normalized number of programming cycles (blue). **b.** Truncated and parent distributions for $\sigma_p =$ 0.3.

In conclusion, the analysis of the experimental results in terms of a gaussian distribution truncated by the censoring interval of the program and verify protocol has allowed us to show that there is an intrinsic conductance state centered at G_0. This validates our experimental protocol and confirms that the obtained results can be traced back to the quantum of conductance which only depends on universal constants of nature.

16. Cycle-to-cycle and device-to-device variability

The results reported in the interlaboratory comparison by each participant come from sets of mixed measurements of cycles made in the same device and in several devices. The analysis of variability through the standard deviation of the measurements mixed the variability from cycle-to-cycle (different cycles taken with the same device) and device-to-device (different cycles from various devices). To distinguish these two sources of variability, the results corresponding to several cycles taken in just a single device were compared with the results mixing up several devices. For this purpose, Supplementary Table 3 compares the standard deviation obtained over multiple cycles of the same device that reflects only the cycle-to-cycle variability (measurements performed by NMI 3 and LAB 3), with results obtained by considering measurements performed over different devices. Values of NMI 3 and LAB 3 for a "single device" were not used in the interlaboratory comparison overall results, but only in the following comparison with the results obtained from multiple devices (note that "single device" values are a subset of the participants' values presented for multiple devices).

For the cases G_1-NMI 3 and G_2-LAB 3, the standard deviation has a similar value, 0.20 G_0 and 0.21 G_0, respectively. In the other case G_1-LAB 3 and G_2-NMI 3, the standard deviation values decreased slightly to 80 % and 88 %, respectively, of the standard deviation for the total number of cycles. It is worth noticing that for all the cases, the difference between the corresponding mean values is fully covered by the combined standard uncertainties of the mean which shows that the random process still to be estimated adequately by the calculated standard deviations.

Comparing the observed values of 0.20 G_0 for G_1 and 0.21 G_0 for G_2 with the other values of the table obtained by the other participants, we can find a comparative base where the measurements result from a higher number of different devices. For example, the 0.20 G_0 could be compared with 0.23 G_0 from NMI 1 obtained from 10 devices and the 0.21 G_0 could be compared with the 0.24 G_0 from NMI 2 obtained from 12 devices or the same value 0.24 G_0 of LAB 1 obtained from 8 devices.

We can conclude that small differences (13 % and 11 %) are observed between the variability of quantum conductance values obtained from measurements where both random effects of cycle-to-cycle and device-to-device are present, and the measurements are mainly affected by the cycle-to-cycle effect. This means that the dominant effect of variability is related to stochastic effects during the formation of the conductive filament.

Supplementary Table 3 | Evaluation cycle-to-cycle and device-to-device variability.

Participant	Mean value $\bar{G}_j$ / G0	Std. Dev. S / G0	Nbr. of values N	Nbr. of devices
G_1				
NMI 1	0.98	0.23	19	10
NMI 2	0.98	0.23	30	10
NMI 3	1.04	0.20	18	2
NMI 3 (single device)	1.02	0.20	15	1
LAB 1	0.95	0.29	16	10
LAB 2	0.97	0.25	15	5
LAB 3	0.88	0.19	19	3
LAB 3 (single device)	0.83	0.15	16	1
G_2				
NMI 1	2.05	0.25	28	9
NMI 2	1.96	0.24	24	12
NMI 3	2.07	0.20	12	2
NMI 3 (single device)	2.13	0.17	9	1
LAB 1	1.94	0.24	10	8
LAB 2	2.00	0.29	14	4
LAB 3	1.99	0.21	12	3
LAB 3 (single device)	2.05	0.21	8	1

17. On the effect of temperature on quantum conductance levels

According to the canonical Landauer theory, electrical transport through the constriction is ballistic and does not involve power dissipation and, thus, is not affected by temperature. Therefore, in principle, we do not lose traceability in the physical observable (i.e. conductance) by changing temperature, even if the measurement protocol to program the cell in the desired quantum conductance level is expected to require adaptation due to different electrochemical dynamics leading to the formation of the filament. Although the conduction of the constriction is expected not to be influenced by temperature, it should be remarked that the dissipation related to inelastic processes in electronic transport can arise at the electrodes (i.e. where electrons thermalize). However, in nanoscale systems like ours, it is difficult to spatially separate constriction and reservoirs. It has been recently shown that there is an asymmetry in the dissipation when a quantum conducting channel opens and that this event generates a thermal gradient which increases the temperature of the gap in a memristor by only a few degree Kelvins.[57] After the formation of the channel, this asymmetry disappears, and power is dissipated in the vicinity of the constriction. In this context, it is worth remarking that power dissipation in quantum point contacts is still an open issue (refer, for example, to ref.[58]). In any case, quantum thermal effects are expected in the adiabatic limit of very low voltages/currents and low temperatures, where ionic motion is essentially suppressed, and only electronic conduction takes place. This is certainly the case at the low voltage (10mV) used to read the conductance state. When higher voltages are applying to modify the filament structure, both electronic conduction and ionic motion are relevant. In this regime, without knowing the exact location where power is dissipated (the constriction itself or the nearby reservoirs), an increase of the filament temperature is expected in the constriction. This effect has been considered in the model of temperature acceleration of the RESET process as presented in Supplementary Section 9.

Supplementary References

1. BIPM - Bureau International des Poids et Mesures. *The International System of Units (SI Brochure) [9th Edition]. SI brochure - Appendix 2* www.bipm.org (2019).
2. Jeckelmann, B. & Jeanneret, B. The quantum Hall effect as an electrical resistance standard. *Meas Sci Technol* **14**, 1229–1236 (2003).
3. Goebel, R., Fletcher, N., Rolland, B., Götz, M. & Pesel, E. Final report on the on-going comparison BIPM.EM-K12: Comparison of quantum Hall effect resistance standards of the PTB and the BIPM. *Metrologia* **51**, 01011–01011 (2014).
4. Goldstein, B. *et al. NIST on a Chip Program Overview*. https://www.nist.gov/system/files/documents/2019/04/02/noac_overview_march_2019.pdf (2019).
5. European Metrology Network on Quantum Technologies. *Strategic Research Agenda*. https://www.euramet.org/european-metrology-networks/quantum-technologies/strategy/strategic-research-agenda.
6. Tsuruoka, T., Hasegawa, T., Terabe, K. & Aono, M. Conductance quantization and synaptic behavior in a Ta 2O 5-based atomic switch. *Nanotechnology* **23**, (2012).
7. Wagenaar, J. J. T., Morales-Masis, M. & Van Ruitenbeek, J. M. Observing quantized conductance steps in silver sulfide: Two parallel resistive switching mechanisms. *J Appl Phys* **111**, (2012).
8. Li, Y. *et al.* Conductance Quantization in Resistive Random Access Memory. *Nanoscale Res Lett* **10**, 420 (2015).
9. Zhu, X. *et al.* Observation of conductance quantization in oxide-based resistive switching memory. *Advanced Materials* **24**, 3941–3946 (2012).
10. Mehonic, A. *et al.* Quantum conductance in silicon oxide resistive memory devices. *Sci Rep* **3**, 1–8 (2013).
11. Chen, C. *et al.* Conductance quantization in oxygen-anion-migration-based resistive switching memory devices. *Appl Phys Lett* **103**, 043510 (2013).
12. Terabe, K., Hasegawa, T., Nakayama, T. & Aono, M. Quantized conductance atomic switch. *Nature* **433**, 47–50 (2005).
13. Gao, S. *et al.* Conductance quantization in a Ag filament-based polymer resistive memory. *Nanotechnology* **24**, 335201 (2013).
14. Younis, A., Chu, D. & Li, S. Voltage sweep modulated conductance quantization in oxide nanocomposites. *J. Mater. Chem. C* **2**, 10291–10297 (2014).
15. Krishnan, K., Muruganathan, M., Tsuruoka, T., Mizuta, H. & Aono, M. Highly Reproducible and Regulated Conductance Quantization in a Polymer-Based Atomic Switch. *Adv Funct Mater* **27**, 1605104 (2017).
16. Song, M. *et al.* Self-Compliant Threshold Switching Devices with High On/Off ratio by Control of Quantized Conductance in Ag Filaments. *Nano Lett* **23**, 2952–2957 (2023).

17. Banerjee, W. & Hwang, H. Quantized Conduction Device with 6-Bit Storage Based on Electrically Controllable Break Junctions. *Adv Electron Mater* **5**, 1900744 (2019).

18. Tsuruoka, T. *et al.* Effects of moisture on the switching characteristics of oxide-based, gapless-type atomic switches. *Adv Funct Mater* **22**, 70–77 (2012).

19. Valov, I. & Tsuruoka, T. Effects of moisture and redox reactions in VCM and ECM resistive switching memories. *J Phys D Appl Phys* **51**, 413001 (2018).

20. Nandakumar, S. R., Minvielle, M., Nagar, S., Dubourdieu, C. & Rajendran, B. A 250 mV Cu/SiO2/W Memristor with Half-Integer Quantum Conductance States. *Nano Lett* **16**, 1602–1608 (2016).

21. Tappertzhofen, S. *et al.* Modeling of Quantized Conductance Effects in Electrochemical Metallization Cells. *IEEE Trans Nanotechnol* **14**, 505–512 (2015).

22. Maudet, F., Hammud, A., Wollgarten, M., Deshpande, V. & Dubourdieu, C. Insights on the variability of Cu filament formation in the SiO_2 electrolyte of quantized-conductance conductive bridge random access memory devices. *Nanotechnology* **34**, 245203 (2023).

23. Deswal, S., Malode, R. R., Kumar, A. & Kumar, A. Controlled inter-state switching between quantized conductance states in resistive devices for multilevel memory. *RSC Adv* **9**, 9494–9499 (2019).

24. Zhao, X. *et al.* Natural Acidic Polysaccharide-Based Memristors for Transient Electronics: Highly Controllable Quantized Conductance for Integrated Memory and Nonvolatile Logic Applications. *Advanced Materials* **33**, 2104023 (2021).

25. Tappertzhofen, S., Valov, I. & Waser, R. Quantum conductance and switching kinetics of AgI-based microcrossbar cells. *Nanotechnology* **23**, 145703 (2012).

26. Xue, W. *et al.* Recent Advances of Quantum Conductance in Memristors. *Adv Electron Mater* **5**, 1800854 (2019).

27. Chen, Q. *et al.* Controlled Construction of Atomic Point Contact with 16 Quantized Conductance States in Oxide Resistive Switching Memory. *ACS Appl Electron Mater* **1**, 789–798 (2019).

28. Xue, W. *et al.* Controllable and Stable Quantized Conductance States in a Pt/HfO_x/ITO Memristor. *Adv Electron Mater* **6**, (2020).

29. Yi, W. *et al.* Quantized conductance coincides with state instability and excess noise in tantalum oxide memristors. *Nat Commun* **7**, 11142 (2016).

30. Long, S. *et al.* Quantum-size effects in hafnium-oxide resistive switching. *Appl Phys Lett* **102**, (2013).

31. Lübben, M., Valov, I., Lübben, M. & Valov, I. Active Electrode Redox Reactions and Device Behavior in ECM Type Resistive Switching Memories. *Adv Electron Mater* **5**, 1800933 (2019).

32. Wedig, A. *et al.* Nanoscale cation motion in TaOx, HfOx and TiOx memristive systems. *Nat Nanotechnol* **11**, 67–74 (2015).

33. Mu, J. *et al.* Application of electrochemical polishing in surface treatment of additively manufactured structures: A review. *Prog Mater Sci* **136**, 101109 (2023).

34. Kim, U. S. & Park, J. W. High-Quality Surface Finishing of Industrial Three-Dimensional Metal Additive Manufacturing Using Electrochemical Polishing. *International Journal of Precision Engineering and Manufacturing-Green Technology* **6**, 11–21 (2019).

35. Ielmini, D., Nardi, F. & Cagli, C. Physical models of size-dependent nanofilament formation and rupture in NiO resistive switching memories. *Nanotechnology* **22**, 254022 (2011).

36. Long, S. *et al.* Voltage and Power-Controlled Regimes in the Progressive Unipolar RESET Transition of HfO2-Based RRAM. *Sci Rep* **3**, 2929 (2013).

37. Benz, S. P. & Hamilton, C. A. Application of the Josephson effect to voltage metrology. *Proceedings of the IEEE* **92**, 1617–1629 (2004).

38. Wagenaar, J. J. T., Morales-Masis, M. & Van Ruitenbeek, J. M. Observing quantized conductance steps in silver sulfide: Two parallel resistive switching mechanisms. *J Appl Phys* **111**, 14302 (2012).

39. Zhu, X. *et al.* Observation of conductance quantization in oxide-based resistive switching memory. *Advanced Materials* **24**, 3941–3946 (2012).

40. Mehonic, A. *et al.* Quantum Conductance in Silicon Oxide Resistive Memory Devices. *Scientific Reports 2013 3:1* **3**, 1–8 (2013).

41. Nandakumar, S. R., Minvielle, M., Nagar, S., Dubourdieu, C. & Rajendran, B. A 250 mV Cu/SiO2/W Memristor with Half-Integer Quantum Conductance States. *Nano Lett* **16**, 1602–1608 (2016).

42. Maudet, F., Hammud, A., Wollgarten, M., Deshpande, V. & Dubourdieu, C. Insights on the variability of Cu filament formation in the SiO2 electrolyte of quantized-conductance conductive bridge random access memory devices. *Nanotechnology* **34**, 245203 (2023).

43. Tappertzhofen, S., Valov, I. & Waser, R. Quantum conductance and switching kinetics of AgI-based microcrossbar cells. *Nanotechnology* **23**, 145703 (2012).

44. Yi, W. *et al.* Quantized conductance coincides with state instability and excess noise in tantalum oxide memristors. *Nat Commun* **7**, 1–6 (2016).

45. Long, S. *et al.* Quantum-size effects in hafnium-oxide resistive switching. *Appl Phys Lett* **102**, 183505 (2013).

46. Krishnan, K., Muruganathan, M., Tsuruoka, T., Mizuta, H. & Aono, M. Highly Reproducible and Regulated Conductance Quantization in a Polymer-Based Atomic Switch. *Adv Funct Mater* **27**, 1605104 (2017).

47. Chen, C. *et al.* Conductance quantization in oxygen-anion-migration-based resistive switching memory devices. *Appl Phys Lett* **103**, (2013).

48. Younis, A., Chu, D. & Li, S. Voltage sweep modulated conductance quantization in oxide nanocomposites. *J Mater Chem C Mater* **2**, 10291–10297 (2014).

49. Tsuruoka, T., Hasegawa, T., Terabe, K. & Aono, M. Conductance quantization and synaptic behavior in a Ta2O5-based atomic switch. *Nanotechnology* **23**, 435705 (2012).

50. Zhao, X. *et al.* Natural Acidic Polysaccharide-Based Memristors for Transient Electronics: Highly Controllable Quantized Conductance for Integrated Memory and Nonvolatile Logic Applications. *Advanced Materials* **33**, 2104023 (2021).

51. Kharlanov, O. G., Shvetsov, B. S., Rylkov, V. V. & Minnekhanov, A. A. Stability of Quantized Conductance Levels in Memristors with Copper Filaments: Toward Understanding the Mechanisms of Resistive Switching. *Phys Rev Appl* **17**, 054035 (2022).

52. Deswal, S., Malode, R. R., Kumar, A. & Kumar, A. Controlled inter-state switching between quantized conductance states in resistive devices for multilevel memory. *RSC Adv* **9**, 9494–9499 (2019).

53. Koymen, I., De Carlo, I., Fretto, M. & Milano, G. Quantum Conductance and Temperature Effects in Titanium Oxide-Based Memristive Devices. *IEEE Trans Electron Devices* **71**, 1872–1878 (2024).

54. Xue, W. *et al.* Controllable and Stable Quantized Conductance States in a Pt/HfOx/ITO Memristor. *Adv Electron Mater* **6**, 1901055 (2020).

55. Banerjee, W., Hwang, H., Banerjee, W. & Hwang, H. Quantized Conduction Device with 6-Bit Storage Based on Electrically Controllable Break Junctions. *Adv Electron Mater* **5**, 1900744 (2019).

56. Chen, Q. *et al.* Controlled Construction of Atomic Point Contact with 16 Quantized Conductance States in Oxide Resistive Switching Memory. *ACS Appl Electron Mater* **1**, 789–798 (2019).

57. Miranda, E. & Suñé, J. Mesoscopic Theory of Resistive Switching. *IEEE Electron Device Letters* **45**, 2029–2032 (2024).

58. Blaas-Anselmi, C., Helluin, F., Jalabert, R., Weick, G. & Weinmann, D. Asymmetric power dissipation in electronic transport through a quantum point contact. *SciPost Physics* **12**, 105 (2022).
